# Beyond Mechanical Recycling: Giving New Life to Plastic Waste

**DOI:** 10.1002/anie.201915651

**Published:** 2020-06-25

**Authors:** Ina Vollmer, Michael J. F. Jenks, Mark C. P. Roelands, Robin J. White, Toon van Harmelen, Paul de Wild, Gerard P. van der Laan, Florian Meirer, Jos T. F. Keurentjes, Bert M. Weckhuysen

**Affiliations:** ^1^ Inorganic Chemistry and Catalysis Debye Institute for Nanomaterials Science, Utrecht University Universiteitsweg 99 3584 CG Utrecht The Netherlands; ^2^ The Netherlands Organisation for Applied Scientific Research (TNO) Delft The Netherlands; ^3^ The Netherlands Organisation for Applied Scientific Research (TNO) Materials Solutions Department Eindhoven The Netherlands; ^4^ The Netherlands Organisation for Applied Scientific Research (TNO) Climate, Air & Sustainability Department Utrecht The Netherlands; ^5^ Energieonderzoek Centrum Nederland (ECN)- part of TNO, Biomass & Energy Efficiency Petten The Netherlands; ^6^ University of Twente Department of Energy Innovation Enschede The Netherlands

**Keywords:** catalysis, chemical recycling, circularity, plastic waste, solvolysis

## Abstract

Increasing the stream of recycled plastic necessitates an approach beyond the traditional recycling via melting and re‐extrusion. Various chemical recycling processes have great potential to enhance recycling rates. In this Review, a summary of the various chemical recycling routes and assessment via life‐cycle analysis is complemented by an extensive list of processes developed by companies active in chemical recycling. We show that each of the currently available processes is applicable for specific plastic waste streams. Thus, only a combination of different technologies can address the plastic waste problem. Research should focus on more realistic, more contaminated and mixed waste streams, while collection and sorting infrastructure will need to be improved, that is, by stricter regulation. This Review aims to inspire both science and innovation for the production of higher value and quality products from plastic recycling suitable for reuse or valorization to create the necessary economic and environmental push for a circular economy.

## Graphical abstract

**Plastic fantastic**: Plastic can rise again and again as a new product. Researchers now know methods with which new plastics can be produced from 100 % recycled material, products that can even be used for food applications. This development is possible thanks to chemical recycling, through which polymer chains are first broken to then be reformed into new molecules, such as plastics but also other chemicals.

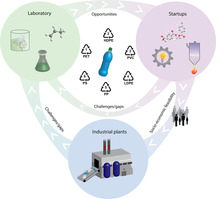

## 1 General Introduction

### 1.1 Introduction

An increase in plastic use (e.g. in everyday products) has assisted in the rapid economic growth of many economies over the last decades. This is in part due to the fact that plastic materials are typically lightweight, have tunable properties and can easily be shaped, thus lending them to a wide range of applications (e.g. automotive, packaging, and housing). However, durability makes plastics an increasing problem for the environment. In contrast to various forms of biomass, such as lignin and chitin, plastic cannot easily be broken down by microorganisms and leads to environmental pollution. Environmental problems are caused by large plastic pieces when ingested by animals as well as by micro‐ and nanoplastics that have an uncertain impact on human and ecosystem health and are present in all our surroundings.^1^ The replacement of plastics will not necessarily have a positive impact on the environment as alternative packaging materials, such as glass or metal containers, are much heavier and increase CO_2_ emissions during transport. The 260 megatons of plastic waste^2^, ^3^ produced annually are detrimental for ecosystems when released to the environment and contributes to climate change because CO_2_ is emitted during incineration. The mass of CO_2_ emissions amount to three times the mass of plastic incinerated. Hence, plastic waste embodies potential CO_2_ emissions equal to 2 % of current global emissions (37.5 gigatons CO_2_ in 2018).^4^ However, plastics also present a great opportunity if the economic value of all these materials can be maintained.

This point is also relevant in the context of fossil‐fuel viability going forward of which 6 % are currently used to produce plastic.^3^ This number is expected to increase strongly due to decarbonization of the energy and mobility sector, while the demand for plastics is increasing due to increasing wealth and urbanization globally. A 2018 report by McKinsey for example states that “*plastics reuse and recycling could generate profit‐pool growth of as much as $60 billion for the petrochemicals and plastics sector*”.^2^

While plastic prices were low and economic incentives for recycling were lacking in the past years, China has recently begun rejecting plastic waste from abroad and it is expected that many other countries like Malaysia will follow soon. Regarding current legislative encouragement for recycling, a legally binding directive of the European Union (EU) states that all plastic packaging shall be recyclable in a cost‐effective manner or reusable by 2030 and aims at making recycling profitable for businesses.^5^ Such legal drivers will push governmental bodies as well as industry to address plastic recycling, ideally in a manner which is circular and enables value creation. Other initiatives are ongoing, including national and European Plastic Pacts, Alliance to End Plastic Waste and New Plastic Economy by the Ellen MacArthur foundation calling for reduce, increased reuse and recycling to tackle the global challenges and moving towards circular plastics economy.

As mechanical recycling is typically accompanied by degraded plastic properties, alternative ways to recycle plastic need to be found. Many reports have highlighted approaches to the conversion of plastic waste to produce syngas or fuels and naphtha.^2^ This Review does not cover gasification routes leading to syngas, but mainly focuses on (catalytic) pyrolysis towards the production of monomers and oligomers as well as solvolytic ways to obtain them. This is with the aim of a circular economy in mind, in which monomers and oligomers can be re‐polymerized following purification and used in the same or similar applications as the virgin polymer equivalent, which is produced from fossil fuels. Dissolution/precipitation and upcycling techniques as well as emerging chemical recycling technologies are also presented as such approaches are crucial in moving towards a circular economy for plastics, supporting an increase above the current 12 wt % of globally recycled plastic, whilst also providing solutions where mechanical recycling is not possible, such as for foils, contaminated and mixed plastic waste streams as well as multilayer packaging products.^3^

### 1.2 Overview of Published Review Articles

A selection of Review articles is summarized in Table 1 (see Table S1 in the Supporting Information for a more extensive list of papers) providing an overview of the main processes analyzed in this Review, sorted by the different processes for easy reference, along with the key messages from past research. Most notable from this summary is the missing link between research and industrially implemented processes as well as the prior focus on fuel and monomer recovery. Complimentarily, a life‐cycle analysis (LCA) is included which assesses the energy requirements and environmental impacts of selected low technology readiness level (TRL) processes—important parameters for a successful economic and ecological commercialization. Another consideration is the mismatch between the purity of researched waste streams and waste streams actually available in current circulation. It is clear that a concerted effort from the entire plastic industry is required to achieve (e.g. EU) plastic recycling targets, including polymer manufacturers, recyclers, legislators, researchers and consumers.

**Table 1:** Overview of a selection of Reviews covering various methods for waste plastic processing.

Title: (published date/ first available online)	Process:	Key messages:
Mechanical and chemical recycling of solid plastic waste^6^ (November 2017)	Mechanical, Pyrolysis	‐ Overview over both mechanical and chemical recycling methods with comparison of limitations, advantages and disadvantages of the different processes ‐ Degradation during mechanical recycling limits closed‐loop recycling although mitigated through stabilizers and compatibilizers ‐ Design For Recycling and From Recycling are important in realizing a circular economy for plastic ‐ Cl and N in waste stream deactivate catalysts in addition to inorganic components blocking pores ‐ Overview and analysis provided of various commercial projects and their status
Solvent‐based separation and recycling of waste plastics: A review^7^ (June 2018)	Dissolution	‐ Gives details of strong and weak solvents for the various polymer types. ‐ Solvent extraction from recycled polymer can cause damage to the polymer chain due to thermal stress. ‐ Dissolution of mixed polymer streams results in poorer separation of the target polymer. ‐ Future use of hazardous solvents should be reduced
PET Waste Management by Chemical Recycling: A Review^8^ (September 2008)	Solvolysis	‐ Polyethylene terephthalate (PET) polymer is difficult to purify once formed, so recycling needs to yield a very pure monomer to allow for repolymerization ‐ Large variety of PET available due to differing degrees of crystallinity ‐ Risks that legislation aims at eliminating polymers that have highest potential for recycling, like PET
Chemical recycling of waste plastics for new materials production^9^ (June 2017)	Solvolysis, Pyrolysis	‐ Hurdles to commercialization are financial incentives and catalyst effectiveness ‐ Unique issues with each type of plastic highlighting the importance of reducing mixed polymer plastics ‐ Progress in design for recycling of polymers will facilitate chemical recycling
Thermochemical routes for the valorization of waste poly‐olefinic plastics to produce fuels and chemicals. A review^10^ (January 2017)	Pyrolysis	‐ Reactor design and process conditions crucial for tuning product distribution due to heat and mass transfer limitations in processing waste plastic ‐ Importance of (acid) catalyst for reducing reaction temperatures

### 1.3 Scope of the Review

This Review highlights more recent innovations and unconventional ideas, which could help solve some of the outstanding issues or even replace existing processes that already received more research focus. We also provide an assessment where research attention is needed. This is performed in three ways. To set the scene, a vision of a circular plastic production process chain is proposed based on utilizing waste plastic instead of fossil carbon feedstocks (Section 1.4.1). Over 150 start‐up companies and large plastic manufacturers who are already working on components of this vision are highlighted. We explain how these companies could make this vision a reality and where more effort will be needed. We conducted interviews with selected start‐ups to discuss experienced barriers to technical implementation as well as the need for policy frameworks. Secondly, the plastics/polymers streams that have the highest potential to be adopted in closed loop or high value chemical recycling schemes are analyzed with regard to environmental and economic impacts through an LCA (Section 1.4.2). This Section establishes a ranking of available recycling technologies, in terms of their potential for CO_2_ emission reduction compared to landfilling and incineration as well as incineration with energy recovery. Finally, text‐mining has been applied to existing literature databases with the aim to analyze which processes and plastics and in which combination have received the most research attention up until the time of writing (Section 1.4.3).

### 1.4 Assessment of the Most‐Promising Areas for Future Research

#### 1.4.1 Role of Chemical Recycling in a Circular Economy

The fraction of collected household plastic waste is between 41 and 76 wt % in France, Germany, UK, Spain, and Italy.^11^ This plastic can be sorted into main streams of polypropylene (PP), high‐density polyethylene (HDPE), low‐density polyethylene (LDPE), PET and polystyrene (PS) using a series of rotary screen drums, near‐infrared sorting and washing steps. This leads to granulates that can be recycled mainly mechanically into non‐food‐grade plastic products, such as flowerpots, paint buckets or shampoo bottles. Polyvinylchloride (PVC) and polyamides (PA) are contaminants. This results in recycling rates of 21 to 42 wt % in the aforementioned countries including exports for recycling to other countries.^11^, ^12^ The actual use of recycled plastic in new plastic products is only 12.3 wt % in Germany.^13^ The collection and recycling schemes of these countries are forerunners while globally 40 wt % are landfilled and 32 wt % leak into the environment.^3^ Therefore, there is a clear need and demand for dramatic improvements in both collection and recycling, also because the shipping of waste to other countries without proper waste management infrastructure for processing raises ecological and ethical concerns.

**Figure 1:**
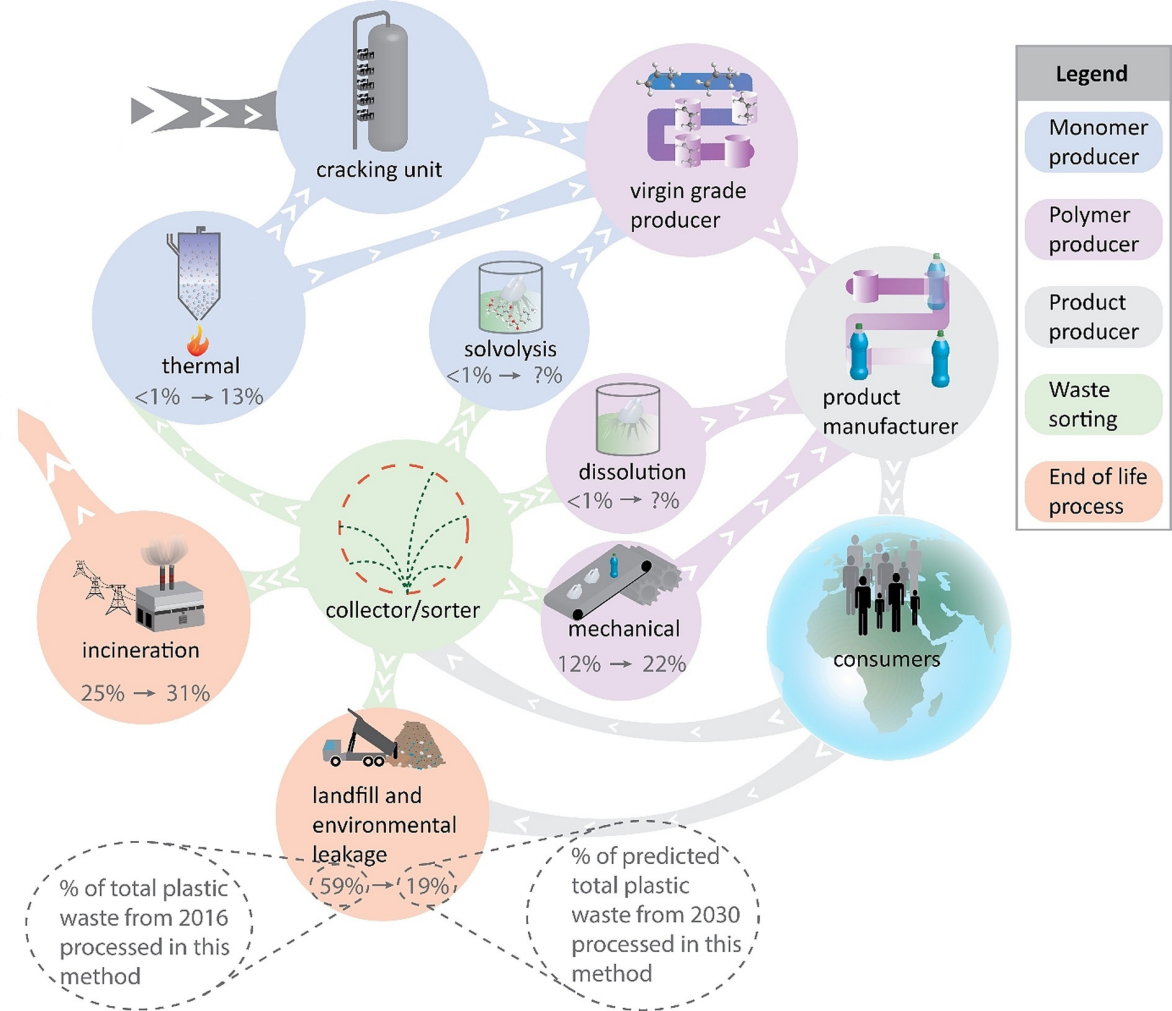
Illustration of an envisioned plastics value‐chain that could enhance the transition to circularity. Currently most plastic is incinerated or landfilled (bottom left), because collection and sorting produce very contaminated and mixed plastic‐waste streams. Better techniques for collection and sorting lead to streams of plastic waste that can be recycled by the various chemical recycling methods. These routes are still going to be complemented by traditional mechanical recycling for the purest steams of a single polymer. The shares that each of the techniques corresponded to in 2016 bottom left of the waste processing method and prediction for 2030 are shown at the bottom right of the waste processing method. These values are based on the McKinsey report.^2^ The plastic objects are sold to the consumer and after its life cycle collected again for sorting to undergo another recycle.

How chemical recycling processes can bolster the circularity of common polymers and avoid landfilling, incineration, and exports to other countries is illustrated in Figure 1. The McKinsey report,^2^ offers an estimate of the size of waste streams in 2030, however dissolution and solvolysis are not mentioned and hence no predictions were included for those processes in Figure 1. Most of these processes are actively developed by companies and start‐ups and we gathered information on these companies, conducting interviews with several of them (Table S9). Some are highlighted here and in Tables S2–S8, whilst a report^14^ lists additional companies specifically regarding polyurethane (PU) recycling.

##### 1.4.1.1 Solvolysis

PET is a special case of an already pure mono‐stream with as little as 16.4 ppm of PVC, 29.4 ppm of other contaminants (Germany) and 4.1 ppm PA (France) and mechanical recycling rates can be as high as 15 wt %.^11^ The recycled resin is, however, mixed with virgin resin to maintain color and structural integrity. Alternatively, very well sorted streams containing only polyesters and polyamides can be depolymerized into monomers by solvolysis (Section 2.2) and used to produce polymer resins by virgin‐grade producers.

Full depolymerization of PET to the monomer bis(2‐hydroxyethyl)terephthalate (BHET) by glycolysis is pursued by Garbo,^15^ IBM,^16^ and Dupont‐Teijin.^17^, ^18^ The purified monomers can be recycled back to PET granulate. Ioniqa,^19^, ^20^, ^21^ operating a 10 kilotons/year facility since 2019, employs ionic liquids to assist glycolysis. Rather than depolymerizing PET all the way to monomers, CuRE^22^ and PerPETual^23^ glycolyze PET chains to low molecular weight (M_w_) oligomers. The oligomers can also be repolymerized to PET granulate. In this way, PerPETual recycles 2 million plastic bottles per day in a plant in Nashik, India and CuRE is planning a pilot plant in 2020. Instead of BHET, hydrolysis of PET yields terephthalic acid (TPA) which is a precursor for BHET. The Infinia process is an example of this and BP has recently announced plans to build a pilot plant based on it.^24^ Efficient heating for alkaline hydrolysis of PET is achieved with a microwave‐reactor in the Demeto‐process operated at the pilot scale since 2014 by Gr3n.^25^, ^26^ Bio‐inspired enzymatic hydrolysis of PET is applied by Carbios.^27^ PA‐6 can also be depolymerized to yield caprolactam. PA‐6 from carpets is hydrolyzed with steam by Aquafil in a plant in Slovenia to obtain pure caprolactam recovered by vapor condensation, which is then recycled back to carpets.^28^ Another solvolysis process, methanolysis is applied by Loop Industries^29^ and in development by Eastman^30^ to produce dimethyl terephthalate (DMT), which can either be used to produce PET directly or via BHET.

##### 1.4.1.2 Dissolution/precipitation processes

The most versatile process for dissolution/precipitation (Section 2.3) was developed by Fraunhofer IVV together with CreaCycle GmbH. Their CreaSolv® process,^31^ which is licensed to other companies, was tested for expanded‐ and extruded‐ polystyrene containing hexabromocyclododecane (HBCD), from waste electric and electronic equipment (WEEE) plastic^32^, ^33^ as well as for packaging. Dissolution/precipitation processes can separate one polymer from mixtures of polymers as present in multilayer films, often PP/PA or PP/PET^34^, ^35^, ^36^ or WEEE plastics.^37^ For example, the NewCycling process, developed by APK AG, can separate the polymers of multilayer films that also contain aluminum foil by stepwise dissolution in methylcyclohexane of PE and PP while increasing temperature according to the Patent.^38^ Since 2018, APK AG runs a commercial plant with a capacities of 8000 megatons/year and plans to build another one with and 25 000 megatons/year capacity in 2020. A special category of separation processes for multilayer films and laminates that use solvents, but do not require full dissolution of the polymers, were developed by Saperatec in Germany and by PVC Separation in Australia. Saperatec plans to address multilayer film separation by reducing the interfacial forces between PET, PE, and aluminum foil in a 18 000 tons/year plant planned to be operational at the end of 2021. The associated Patent indicates use of an organic solvent‐based micro‐emulsion for swelling and a carboxylic acid to accelerate delamination.^39^, ^40^ The Australian company PVC Separation developed a process to delaminate multilayers by swelling the polymer in a low boiling solvent. Exposure to hot water, causes the solvent to flash out and release the desired polymers, which can be separated in a sifter due to density differences.^41^

Dissolution/precipitation processes also allow for the removal of colorants and other additives via filtration to produce higher purity resins, competitive to virgin polymer, and to recover valuable additives. PolyStyreneLoop recycles polystyrene foam demolition waste by removing HBCD not only recovering polystyrene but also elemental bromine, which can be used for the synthesis of new BFRs. The construction of a plant with an annual capacity of 3300 tons input has started in December 2019 in Terneuzen, the Netherlands.^32^, ^42^

The choice of an environmentally benign solvent with high dissolution capacity that can be easily recovered often decides the fate of the development of a dissolution/precipitation process. For example, PVC waste recycling with butanol as solvent and steam as anti‐solvent by VinyLoop was shut down after more than 15 years of operation, because the process was not effective enough to remove additives, such as plasticizers.^43^ However, some innovative solutions are developed. A green high‐boiling aromatic solvent, cymene, derived from citrus fruit industry waste streams is used for recovery of PS from packaging by technology licenser Polystyvert.^44^ The process of PureCycle with a facility under construction dissolves PP in supercritical butane followed by precipitation upon decompression.^45^

##### 1.4.1.3 Upcycling

Producing chemicals from plastic waste that are more valuable than monomers, polymers, or the feedstock of steam‐crackers is an alternative to valorize plastic waste (Section 2.4). BioCellection makes organic acids from PE that are essential in the production of performance materials, such as solvents and coatings.^46^

##### 1.4.1.4 Thermal routes

Pyrolysis (Section 2.5) takes relatively mixed plastic waste streams, but handle only small amounts of other organic, PVC, PU, and PET impurities depending on the process. It typically yields fuel‐like products. In partnership with polymer manufacturers, the pyrolysis oil is further upgraded to produce the monomers for making virgin grade resins (SABIC in partnership with Plastic Energy and Petronas,^47^ Fuenix Ecogy and Dow,^48^ Shell with Nexus Fuels,^49^ BASF with RECENSO GmbH^50^ etc.). These resins can be made into plastic objects by the converter.

Whilst well‐researched, the highly mixed product stream with hydrocarbons spanning various boiling point ranges from diesel to gasoline and waxes limits applicability of pyrolysis in standard reactors, and there is ample room for improvement. OMV addresses problems of heat‐ and mass‐transfer encountered in highly viscous and not very heat‐conductive plastic, dissolving it in a crude oil fraction before feeding it to their 100 kg hr^−1^ test pyrolysis reactor.^51^ Catalytic Tribochemical Conversion developed by RECENSO GmbH attempts to overcome heat‐ and mass‐transfer limitations via mechanical force, which also lowers the reaction temperature required since mechanical energy is introduced in addition to thermal energy.^50^ The process is currently rated at TRL 6/7 and is operated at the pilot plant scale. The Cat‐HTR process operated for 10 years already at the pilot‐plant scale achieves a very homogeneous heat distribution, injecting supercritical water into the plastic waste.^52^ The supercritical water also quenches unwanted side reactions leading to high yields of stable hydrocarbon liquids. This process is insensitive to moisture and contaminations of the plastic with other organic waste. A catalytic process is applied by the Dutch/Indian company Patpert Teknow Systems with 40 installations varying in process capacity between 110 and 7300 tons/year.^53^ The plastic is co‐fed with a silica/alumina based cracking catalysts to the pyrolysis reactor at 350–360 °C.^54^ Heavy wax fractions are separated from other products and fed to a secondary catalytic cracking unit, followed by an integrated fractionation column containing a catalyst fixed bed.

##### 1.4.1.5 The future of chemical recycling

Based on the brief overview and the compiled list of companies (Table S2–S8) active in the field of chemical recycling, it is clear that more selective solvolysis and pyrolysis processes are required and that catalytic processes have not found much application to date. More innovation in the field of upcycling is also required to create more business opportunities. It is interesting to note that pilot plants for solvolysis,^55^ pyrolysis,^56^ and hydrocracking^57^, ^58^ were planned or already existed in the nineties, however when contacting them, a lack of suitable waste streams, logistics and commercial viability were cited as reasons for discontinuing the project or knowledge of these processes was not available anymore. Specifically, the price competition of pyrolysis oil with crude derived petroleum hinders commercial viability. Due to recently renewed interest, a number of new market entrants are at a similar stage of development as in the 1990s. Processes are usually developed in demonstration plants and then licensed out. As crucial in determining the fate of this new wave, contacted companies name the implementation of appropriate policy frameworks, such as a tax on CO_2_ with clear definitions of chemical plastic recycling (Tables S2–S8). A better integration and collaboration of all stakeholders along the value‐chain as well as a more forward‐looking investment strategy, standardization, and legislation are necessary. CO_2_ emission penalization/taxation or other measures that integrate environmental costs, are needed to drive market interest and further adoption of chemical recycling. Likewise, more stringent packaging design regulations that dictate better recyclability and stricter regulations on waste disposal would pave the way towards a circular economy.

#### 1.4.2 Life‐Cycle Analysis of Chemical Recycling Processes and Plastics

Circular end‐of‐life (EoL) treatments aim at reusing the plastic directly or as a resource, for example, through chemical or mechanical recycling, whereas plastics are merely disposed in linear EoL treatments, such as incineration or landfilling. We analyzed the environmental benefit of these EoL treatments in terms of CO_2_ emissions, which arise from resource and energy needs and relate to climate change as well as depletion of fossil resources and reduction in air quality. A discussion of other indicators such as toxicity, eutrophication, and water‐demand requires a more detailed study beyond the scope of this Review.^59^ LCA is performed in reference to one ton of plastic waste having experienced the full life cycle of plastic production, product manufacturing, waste collection, transport, sorting, and recycling also accounting for losses in each of those steps. We benchmark the recycling options and EoL treatments listed below against waste incineration and landfilling:

1. incineration with energy recovery
2. pyrolysis (see also Section 2.5)
3. mechanical recycling
4. solvolysis (see also Section 2.2)
5. dissolution/precipitation (see also Section 2.3)

Chemical recycling could complement mechanical recycling, addressing mixed polymer and composite plastics that can only be recycled to lower quality products. Therefore, we analyzed EoL options for multi‐material plastic waste with diverse applications and meaningful volumes:

1. acrylonitrile butadiene styrene (ABS) from a back panel often used in electronics
2. high‐impact polystyrene (HIPS) from a case used for food products
3. PET from drinking bottles
4. PET‐PE multilayer food packaging foil
5. glass fiber reinforced polypropylene (PP‐GF) from a composite door panel used in transport.

With incineration, 5 to 10 tons of CO_2_ are released by 1 ton of plastic over its life cycle. Variations are mainly caused by differences in carbon content and energy requirements of production or the different plastic types (Tables S10–S11), because melting point, viscosity, tensile strength, and energy content affect process parameters. Approximately half of life‐cycle CO_2_ emissions stem from the plastic production, while less than a third of CO_2_ emissions arise from embodied carbon released by incineration. The remainder is related to final product assembly, amounting to less than 10 wt % for multilayer packaging and around 25 wt % for the composite PP‐GF. Emissions from transport of waste are minimal. Energy recovery avoids 30–45 wt % CO_2_ emissions, which would arise from traditional electricity generation.

**Figure 2:**
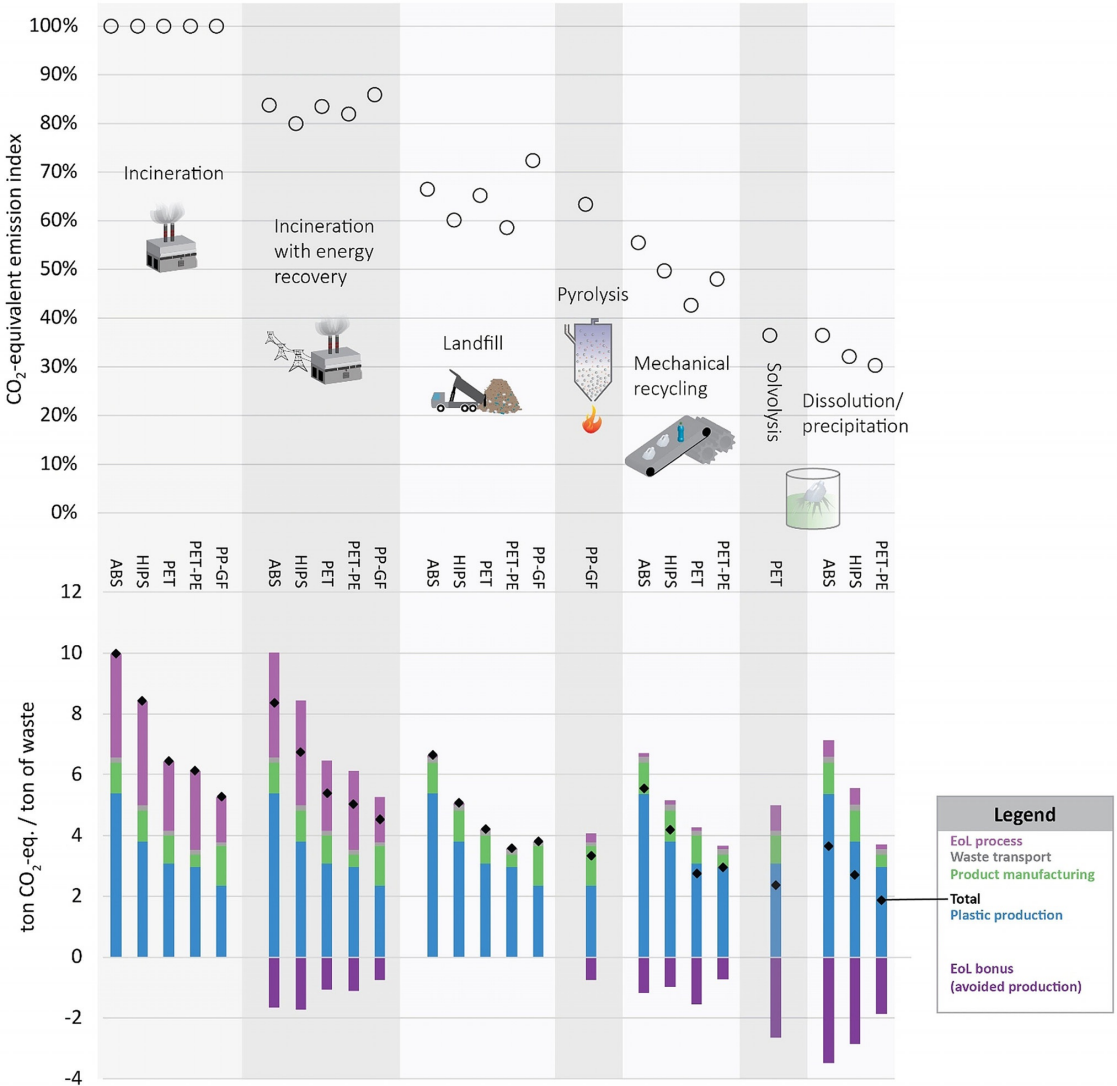
Top: CO_2_‐equivalent emissions of different EoL treatment technologies applied for several plastic‐waste streams, in relative emissions indexed to incineration (100 wt %). Bottom: CO_2_‐equivalent emissions of different EoL treatment technologies in absolute emissions in ton CO_2_/ton waste by life ‐cycle stage.

All alternative treatments to incineration reduce process EoL CO_2_ emissions, since production of a new resource is avoided. CO_2_ emissions of different EoL treatments indexed against incineration without energy recovery are ranked by the potential to save CO_2_ emissions (Figure 2).

In case plastic waste is landfilled, embodied carbon is hardly released over time (<2 wt. %)^60^ which reduces life‐cycle CO_2_ emissions to a third compared to incineration without energy recovery. But it has to be noted that landfilling is not circular, requires a lot of land and causes toxic substances to leach into the soil and groundwater unless prevented by costly measures.

For dissolution/precipitation, CO_2_ savings are the highest at 65–75 wt %, because no bonds are broken or need to be reformed. With solvolysis similar amounts of CO_2_ can be saved due to an efficient conversion into a high‐value recyclate, possible, for example, for uncontaminated PET waste. Pyrolysis products can replace fuel oil and natural gas which avoid 30 wt % of the CO_2_ emitted during incineration. Mechanical recycling of mixed plastics with additives only avoids around 25 wt % of CO_2_ emissions due to the low recyclate quality.

CO_2_ savings only come from avoided plastic, pyrolysis oil, or energy production. Recycled products still need to be remanufactured and transported, meaning that reuse is advantageous over all recycling routes. However, electrification of these processes via renewable energy supply could be capable of de‐fossilizing process schemes and steps but is not directly related to recycling and thus not included in the analysis. It is also worth noting that future polymer production may look to sustainable monomer sourcing pathways, that is, via CO_2_‐derived methanol, thus reducing emissions profiles still further for certain products.

While the analysis identifies benefits of certain EoL technologies over others, it needs to be extended to more plastic waste streams and chemical‐recycling technologies, including emerging technologies (Section 2.1). However, the results indicate, perhaps logically, that the less the polymer structure/bonds are broken and the higher the quality of the recycled product, the better the environmental performance. Moreover, internalizing CO_2_ costs in the product price could make circular options more profitable than polymer production from fossil sources. Dissolution/precipitation for example avoids between 3 to 6 tons CO_2_ for each ton of plastic waste. If this CO_2_ were to be priced at 50 to 200 Euro/ton, recycled plastic product would have a price advantage of 150 to 120  Euro per ton of plastic waste. This is significant, considering that the typical polymer value is around 1500 Euro/ton.^61^

#### 1.4.3 Analysis of the Most‐Researched Processes and Plastics

We analyzed 474 relevant research articles by using a custom text‐mining script using the Scopus application programming interface (API) to understand which chemical recycling processes have been used for which type of plastic and how much research attention they have received (Figure S1). Surprisingly, the most researched plastics are not the plastics produced in the highest volume. PET is by far the most researched plastic type, appearing more than 2000 times in the searched literature. This is presumably because collection systems are already in place for PET, raising awareness in the public. The availability of plastic waste (mono‐)streams seemingly dictates research effort. In contrast, HDPE having twice the share of plastics production^14^ compared to PET only appeared 353 times. It is generally more energy intensive to break the strong C−C bonds of polyolefins selectively compared to the ester bonds in PET, which can be depolymerized at relatively mild conditions with high selectivity to monomers.

Pyrolysis is by far the most mentioned process and often appears together with cracking. It can, however, be seen that catalytic pyrolysis and cracking have received far less attention, although catalytic pathways can greatly influence the recyclate product scope (Section 2.5). Another interesting process that remains under‐researched is hydrocracking (Section 2.5.3). Finally, very little research has focused on real waste streams, such as plastic solid waste (PSW) and municipal plastic waste (MPW).

## 2 Problems and Novel Solutions

### 2.1 Emerging Technologies

**Figure 3:**
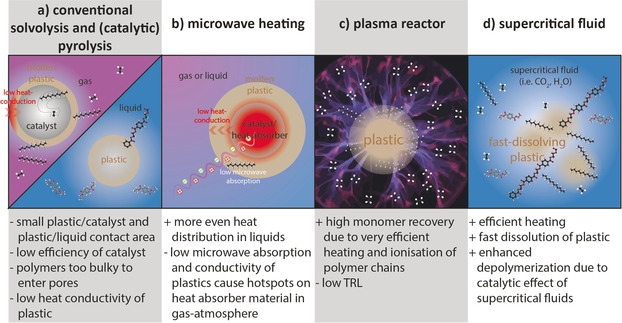
Microwave heating (b), plasma reactors (c) and supercritical fluids (d) can address some of the problems encountered in conventional solvolysis and pyrolysis (a).

Several unconventional approaches to polymer recycling are discussed in the literature. Some use alternative ways to supply energy for the depolymerization as in mechanochemical approaches, photo‐reforming as well as microwave and plasma reactors. Bio‐inspired routes include polymer digestion by enzymes. There are also efforts to use alternative solvents, such as supercritical fluids, or ionic liquids (ILs) and deep eutectic solvents. These approaches attempt to address problems encountered in conventional pyrolysis and solvolysis (Figure 3 a), namely low heat conductivity of plastic and general heat distribution problems in industrial reactors; and the issue of a relatively small contact area between the solid plastic and the reactive media or catalyst. The methods described in Figure 3 b–d have the potential to achieve more efficient heating, higher conversion and higher selectivity.

Conversion under microwave irradiation (Figure 3 b) and in supercritical fluids (Figure 3 c) often lead to more homogenous temperature profiles throughout the reactor. This can lead to faster heating rates and a more controlled and selective reaction, due to similar reaction rates at all locations of the reactor. Reactions are also sped‐up in several reported cases. For example, microwave reactors can lead to shortened reaction times during solvolysis, but the use of a catalyst is required to achieve high selectivity to monomers.^62^, ^63^ This approach is discussed in more detail in Section 2.2. This rather novel way of reactor heating was also applied to pyrolysis, discussed in Section 2.5.

Solvolysis in supercritical fluids is also compared to conventional solvolysis approaches in Section 2.2. In contrast to normal solvolysis conditions, supercritical fluids (e.g. H_2_O) can also depolymerize polyolefins, which have a chemically very inert backbone that is harder to cleave than ester and acid amide bonds present in PET and PA respectively. By tuning the applied temperature and pressure of the supercritical fluids, such as, alcohol and water, solvation (as well as basic/acid properties for water) can be altered, making them catalytically active.^64^, ^65^ This was reported for treatment of composites such as fiber reinforced plastic.^66^

Under supercritical conditions (i.e. H_2_O) even polyolefins can be depolymerized. The C−C bonds in the polyolefin backbone are harder to break that ester or acid amide bonds in PET and PA. This is often called pyrolysis under supercritical conditions. However, in comparison to pyrolysis (typically performed under an inert atmosphere), supercritical water or alcohol are not inert and thus hydrogenated or oxygenated products are formed during treatment.^66^ An advantage of supercritical fluid treatment of polyolefins is the relative insensitivity to organic impurities and moisture usually present in PSW as demonstrated by the Cat‐HTR process.^52^, ^67^ The use of plasma‐assisted pyrolysis, which often leads to higher monomer yields is discussed in further detail in Section 2.5.

ILs, as with supercritical fluids, address the problem of the small contact area between the plastic and the reactive media and/or catalysts in conventional solvolysis and pyrolysis. Use of ILs for solvolysis is discussed in Section 2.2. ILs are already applied commercially for other processes. For example, the BASIL process by BASF to scavenge acids in the synthesis of alkoxyphenylphosphines was commercialized in 2002 and has shown to be much more environmentally friendly than the previous process using tertiary amines.^68^, ^69^ It was found that with ILs, PE can be cracked exclusively to alkanes in yields of up to 95 % at below 250 °C although relatively slowly.^70^ While ILs are often used to replace organic solvents (they have negligible vapor pressure) and can act themselves as catalysts (Section 2.2 and 2.3) they suffer from high toxicity, poor biodegradability and often high costs.^71^ A proposed alternative are eutectic solvents. These are two solids, which when combined have a low melting point and can act as acid or base through functionality choice.^72^ This class of solvents has been used for PET glycolysis leading to high selectivity to the monomer BHET.^73^

#### 2.1.1 Mechanochemistry

Drawing inspiration from the field of polymer manufacture, the application of mechanical stress has been shown to lead to the homolytic cleavage of polymers and thus radical formation. This can lead to cross‐linking and cross‐polymerization, and can restore the properties of plastic, but it can also be used to promote depolymerization.^74^, ^75^, ^76^, ^77^, ^78^, ^79^, ^80^, ^81^, ^82^, ^83^, ^84^, ^85^, ^86^ No additional heating is required, because the necessary heat is generated at the ball impact zone.

Mechanical stress can occur in shear‐reactors, ball mills, as well as during sonication (e.g. imploding bubbles). The depolymerization of poly(phthalaldehyde) was achieved by imploding cavitation bubbles formed during sonication. A *M*_w_ dependence was observed. When starting with high average *M*_w_ polymers (458 kg mol^−1^) depolymerization was faster than for lower *M*_w_ fractions (<26 kg mol^−1^).^82^ Sonication was performed under Ar at a low temperature (−40 °C; *t*=6 h) to counteract heat generation, leading to 60 % depolymerization in the high *M*_w_ case. This highlights a limitation of mechanochemical bond scission. There is a limiting *M*_w_, under which the chemical energy generated by the applied mechanical force is not sufficiently accumulated to break covalent bonds.^87^

Mechanochemical effects can also be used to pre‐treat waste plastics, facilitating pyrolysis. For example, the need to remove halogen impurities which cause generation of corrosive and toxic gasses during pyrolysis can be avoided in this way. The energy demanding de‐chlorination of PVC and removal of bromine additives from PS has been achieved using ball milling.^88^ Ball‐milling assisted de‐chlorination of PVC can be performed in the presence of dry CaO, which itself becomes chlorinated and can subsequently be removed by washing.^89^ The process can also be run in wet and basic conditions.^90^ As another pre‐treatment for pyrolysis, a steam‐explosion treatment has been shown to lower the liquefaction temperature by 50 °C for PS and by 100 °C for HDPE.^91^

#### 2.1.2 Ambient‐Temperature Photo‐Reforming

Regarding the utilization of (direct) photonic energy, Reisner et al. reported on ambient‐temperature photo‐reforming of plastic waste into fuel and bulk chemicals. Cut pieces of raw polymer or fibers of PET and polylactic acid (PLA) with and without food contamination were converted under aqueous alkaline conditions, using an inexpensive, non‐toxic photocatalyst.^92^ Ni_2_P supported on cyanamide‐functionalized C_3_N_4_ was reported to promote efficient charge separation with a photostability of ca. 120 h. This approach represents an elegant, simple and low‐energy transformation of plastic waste to valuable products, which was previously performed with a precious‐metal or Cd‐based photocatalysts.^93^

#### 2.1.3 Biotechnology

Nature's own catalysts, enzymes and enzymatic collectives in cells and microorganisms, have been shown to able to degrade plastic waste.^94^, ^95^ Enzymatic hydrolysis of polyesters, that is PET, can be used both to functionalize the surface of the plastic and to fully depolymerize it.^96^ Degradation of several plastics by microbial enzymes was reviewed by Wei et al.^97^ and the recently published Plastics Microbial Biodegradation Database (PMBD) features a list of 949 microorganisms–plastics relationships referring to 79 specific genes that are known to be related plastic biodegradation.^98^ Biodegradation pathways relate to chemical “familiarity” and enzymes act on chemical bonds that are found naturally, that is, glycosidic, amide, and ester bonds. With respect to “Design for Recycling” concepts this is an important consideration and makes addition polymers, such as, polyolefins more challenging.

While aliphatic polyesters are known to be enzymatically hydrolyzed even at room temperature, an enzyme for the aromatic polyester PET with appreciable rates was discovered in 2005 by Müller et al.^99^ Many researchers investigated the enzymatic hydrolysis of PET using cutinases and its mutations. The full degradation of PET plastic waste and recovery of monomers was reported using the bacterium, *Ideonella sakaiensis* 201‐F6 discovered near a PET bottle recycling plant and its expressed hydrolases, PETase and MHETase (a mono‐(2‐hydroxyethyl)terephthalate‐digesting enzyme).^100^ The mutation of two active‐site residues of cutinase are responsible for PET degradation back into the monomers: BHET, and TPA.^101^ MHETase is responsible for the further hydrolysis of MHET to TPA and ethylene glycol (EG), which can be used in PET production. Generally, crystalline PET cannot be hydrolyzed by enzymes and enzymatic hydrolysis is thus preferentially performed above glass transition temperature, between 67 and 81 °C depending on the crystallinity. Enzymatic depolymerization of PET and the biodegradable polymer PLA has been commercialized by Carbios. The Patents describe an pre‐amorphization step^102^ and the use of several enzymes.^103^, ^104^ Polyurethane varnish and polyether polyurethane foams were degraded by up to 87 % after 14 days of incubation through a microbial enzyme of a fungi, *C. pseudocladosporioides* strain T1.PL.1.^105^ In addition, enzymes were recently also found to be useful for synthesis of biodegradable plastics.^106^ While not many commercial processes based on enzymes exist to date, further discoveries and advanced protein‐engineering techniques through which further mutants may be accessed might make enzymatic depolymerization a viable technology in the future given the low temperature required and thus small energy requirements.

#### 2.1.4 Design for Recycling

Polyolefin‐based waste has historically been treated via gasification or unselective pyrolysis. There is renewed interest in the conversion of PE and PP back into monomer and/or oligomers. This could be more easily achieved, if the plastics are designed with recycling in mind, for example, by avoiding additives that lead to degradation during melting and re‐extrusion or that cause catalyst deactivation in thermal processes. Mülhaupt et al. discuss this so‐called “design for recycling” approach^107^ for polyolefins.^108^ This Review and other literature from Mülhaupt et al.^109^ discuss the production of 100 % polyolefinic plastics and composites without additives. This can be achieved with multisite polymerization catalysts and specialized injection‐molding processes, such as oscillating packing injection molding. This approach removes the necessity of producing different polymers in different plants, avoids the use of additives, such as glass fiber, and generates a product that could potentially be depolymerized with polymer synthesis catalysts like Ziegler–Natta. The synthesis and production of PP or PE‐based plastics or composites in this manner,^110^ can potentially be more impactful with regard to production efficiency and ecological footprint.

An alternative and inspiring approach for the production of polymers with tunable degradation properties was recently discovered by Shieh et al.^111^ In this study, silyl ether‐ based cyclic olefins were copolymerized with norbornene derivatives to produce copolymers with varying stability when exposed to HCl. Whilst not yet widely applied, the ability to design polymers that degrade under pre‐determined conditions into their monomers (or oligomers) is a key step forward in the recycling of plastics.

### 2.2 Solvolysis

As mentioned in Section 1.4.1, solvolysis is a potentially more selective way to recover monomers from polyesters and polyamides employing lower temperatures compared to those used in pyrolysis. The solvolysis processes hydrolysis, alcoholysis (glycolysis and methanolysis), phosphorolysis, ammonolysis, and aminolysis cleave ether, ester, and acid amide bonds and therefore are limited to polymers with these bonds. Much R&D focusses on PET^112^, ^113^, ^114^, ^115^, ^116^ and PU^117^, ^118^ and to a lesser extent on PA, polycarbonate (PC)^119^ and PLA^120^ depolymerization. The advantage of these processes lies in the possibility to obtain monomers that can be further purified, filtering out additives and colorants, allowing for re‐polymerization to virgin‐grade quality. This is especially interesting for PU, which cannot be recycled mechanically. If the purity or quality of the recovered monomers is not as good as the monomers initially used, they can also be mixed with conventionally obtained monomers for the polymer synthesis. A few commercial plants exist for methanolysis of PET^121^ and glycolysis of PET and PU.^118^ The further development of these processes over hydrolysis were ascribed to higher energy requirements of hydrolysis, because of high operating temperatures needed.^122^, ^123^

**Figure 4:**
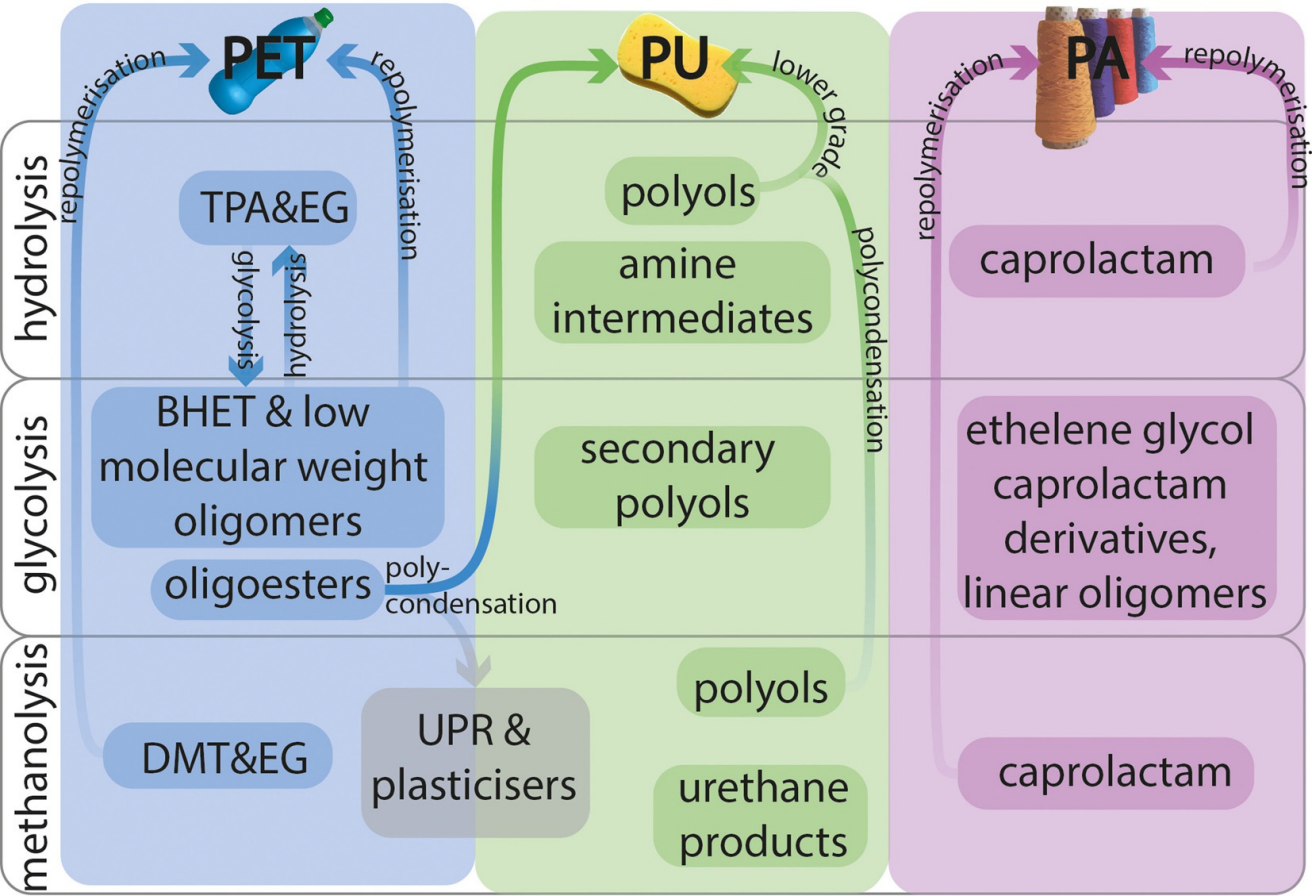
Products obtained through the different solvolysis pathways of PET, PU, and PA and how these products can be used to recycle back to the polymer or to obtain valuable products.

PET can be depolymerized to the monomers TPA, BHET, or DMT, depending on the process (Figure 4). While BHET and DMT can be directly used for the polymerization to PET, TPA needs to be converted into BHET first. Nevertheless, this process has replaced the polymerization from DMT more and more. The BHET that is obtained from glycolysis is not pure enough to produce PET directly and oligomers are produced as well that can be used to synthesize PU or unsaturated polyester resins (UPR).^124^ Thus, hydrolysis which allows the production of high purity TPA appears very attractive. However, high purity BHET can be obtained employing a metal acetate^125^ or sodium carbonate^126^ catalyst in glycolysis, the sodium carbonate produced almost an 80 % yield after 1 h at 196 °C with a reported EG:PET molar ratio of 7.6:1.

In contrast to PET, PU solvolysis often yields other chemicals that cannot be used directly to make the same PU again, but another type of PU.^117^, ^118^ It was discovered that using both water and glycol as cleavage agents, so‐called hydroglycolysis, yielded a more pure polyol.^127^ A further demonstration of hydroglycolysis was to obtain TPA from PET with reduced energy input.^128^ For the hydroglycolysis of PU, however, the higher processing cost associated with separation was noted.

Microwave heating has been developed for more efficient heat delivery. This technique can provide a more even temperature profile inside the reactor, as it heats volumetrically. This leads to faster depolymerization, an example with 1‐pentanol as the solvent utilized microwave heating to reduce the time for complete decomposition of PET using a KOH catalyst from 32 min under normal heating to just 2.5 min.^128^ Microwave assisted glycolysis of PU^129^ and hydrolysis of PA^130^ has also been reported. However, the purity of the product might not be the same as under normal heating and the differences in products are rarely reported in much detail. Microwave assisted non‐catalytic glycolysis of PET lead to a mixture of oligo‐esters with unknown purity.^62^ While in the non‐catalytic microwave hydrolysis of PET, 100 % yield to TPA and EG was achieved over a 120 min reaction.^63^ To understand, whether microwave‐assisted solvolysis is more economical than conventional heating, a detailed comparison of power usage would be beneficial as this was found to constitute the highest operating cost.^131^ In the case of biodiesel production from palm oil, the energy consumption was found to be halved when employing a microwave reactor.^132^ However, since microwave heating is relatively inefficient, the overall power requirements stay similar.^133^ Scaling up was found to have a positive effect on energy efficiency.^134^ The fact that companies like Gr3n^25^, ^26^ and Pyrowave^135^ are currently developing this technology at commercial scale shows the promise of this technology.

Other issues encountered in all solvolysis processes are

1. separation of the liquid cleavage agent and other by‐products,
2. small contact area between the liquid cleavage agent and the solid polymer, and
3. recovery of dissolved catalysts.

Re: 1) Aminolysis, phosphorolysis of PU and if in excess of glycol also glycolysis, lead to phase separation of the products from the solvent. Alternatively, the monomers can be recovered by crystallization, distillation or by liquid extraction, that is, with water. Owing to the high cost of using excess glycol, commercial plants only employ single‐phase glycolysis of PU, which leads to less pure polyols. To improve process economics, de Lucas et al. employed crude glycol, a waste by‐product from the diesel industry, which lead to an even purer product than the best performing glycol EG.^136^

Re: 2) The small contact area between liquid and solid plastic pieces retards the reaction, which is why the reaction proceeds much faster at temperatures exceeding the plastic melting point.^115^ An approach to lower the reaction temperature is reactive extrusion, which also decreases reaction times from hours to minutes.^124^, ^137^ The glycolysis of PET via reactive extrusion led to low molecular weight oligo‐esters,^124^, ^138^ which could be used to make PU. However, more research effort is needed to make this process more selective to BHET. Another approach uses a phase‐transfer catalysts that was shown to transport sodium anions from NaOH to the plastic surface of different nylons and PET in basic hydrolysis.^124^ For PET, the yield increased from 2 to 90 % over the reaction time of 5 h at 80 °C. Organocatalysts, such as the quaternary ammonium salts, are used as phase‐transfer catalysts^139^ and can assist glycolysis of PET via hydrogen bonding.^140^ In addition, performing hydrolysis or methanolysis in supercritical fluids addresses the problem of plastic/cleavage‐agent contact and provides homogenous and fast heating.^66^, ^141^

Re: 3) Catalyst recovery can be addressed using solid‐acid catalysts^142^ and manganese oxide based catalysts for glycolysis of PET.^143^, ^144^, ^145^, ^146^ The yield of BHET using S/Zn‐Ti at 180 °C for 3 h was found to be 72 %, which is close to that achieved using homogeneous catalysts.^142^ While comparison to non‐catalytic glycolysis was not always provided, some demonstrated an increase of reaction rate and higher BHET monomer recovery, especially for high surface area catalysts, such as mesoporous metal oxide spinel catalysts.^143^, ^144^

Another novel approach employs ILs,^147^ which can act as strong solvation agents for polymers.^148^, ^149^ Although applied commercially in a few cases ILs can have a high toxicity and LCA has shown that they only become viable, when separated more efficiently from the reaction product.^150^ When employed with the right counter‐ion, depolymerization products can be separated by liquid–liquid extraction.^151^, ^152^ Distillation however is limited, because most ILs used for depolymerization are not volatile. Non‐neutral ILs, that is, basic 1‐butyl‐3‐methylimidazolium acetate [Bmim][Ac] can act as catalyst and fully glycolyze PET.^153^ However BHET monomer yields typically reported with ILs do not exceed 60 %. Sub‐ and supercritical and depolymerization using ILs is also addressed in Section 2.1.

### 2.3 Dissolution/Precipitation

**Figure 5:**
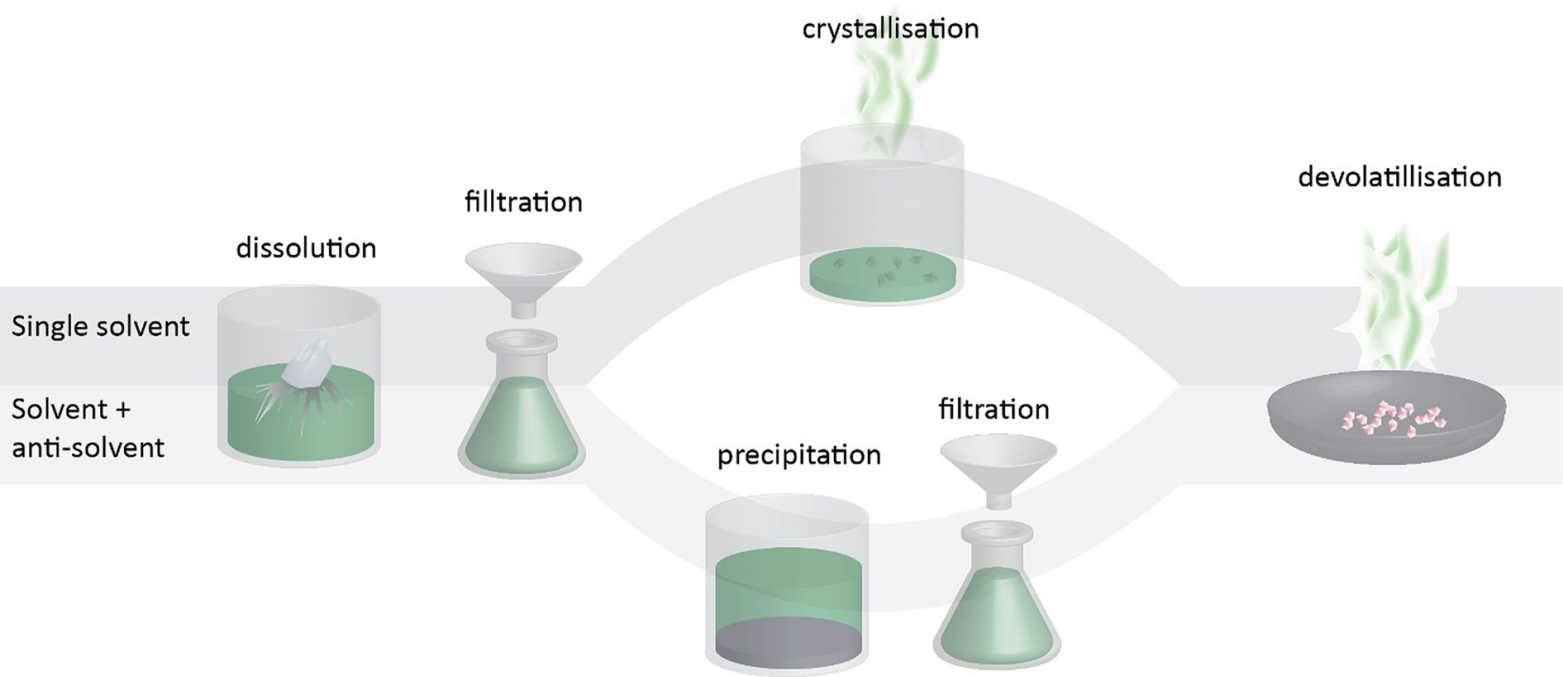
The plastic is dissolved and undissolved fragments, such as pigments, are removed by filtration. The top route describes the dissolution/precipitation technique using a single solvent, which is removed by evaporation, crystallizing the polymer for recovery. For the bottom route an anti‐solvent is used to precipitate the polymer, which can be recovered by filtration. Both routes require a solvent removal step, which can be time and energy consuming unless a supercritical anti‐solvent is employed.

Dissolution/precipitation offers the possibility to recover polymers from plastic waste that are free of additives, such as pigments. Other additives, such as flame retardants, can even be recovered for reuse.^154^ This can be achieved using a single solvent or a combination of a solvent and an anti‐solvent (Figure 5).^7^ For the solvent/anti‐solvent approach, the solvent selectively dissolves a specific polymer, an anti‐solvent is then added to precipitate out the polymer for recovery. In between dissolution and precipitation steps, non‐dissolved materials, such as pigments, are separated from the polymer solution.^155^ The mixture of solvent and anti‐solvent obtained must be separated again for re‐use in the process. Such a separation is energy and time consuming for solvents with a high boiling point.

Another problem is the complete removal of solvents, as any residual solvent affects polymer properties.^156^ Solvent removal and separation is easier if one of the solvents is a supercritical fluid as they readily evaporate when the pressure is decreased. An example of a solvent/anti‐solvent system employs formic acid as a solvent and either supercritical dimethylether^157^ or CO_2_ 158 as anti‐solvent. A further example achieved 97 % extraction of BFR from a typical WEEE plastic, HIPS dissolved in d‐limonene, which is an environmentally friendly solvent.^154^ The extraction was carried out at 65 °C and 20 MPa, with a 2:1 ratio supercritical CO_2_ to d‐limonene. Very few polymers fully dissolve in supercritical CO_2_ 159 and therefore it can be used to remove impurities, such as volatiles, flame retardants, stabilizers and dyes from polyolefins, polystyrene, ABS and PVC, recovered from packaging, WEEE and flooring waste.^160^, ^161^, ^162^ Using supercritical CO_2_ has the added benefit of avoiding toxic organic solvents, such as xylene, acetone, toluene, and *n*‐heptane.^163^

An elegant process for dissolution and precipitation has been reported employing a switchable hydrophilicity solvent (*N*,*N*‐dimethylcyclohexylamine) that dissolves LDPE in its non‐polar form at elevated temperate. Upon cooling of the solution and with the addition of CO_2_ and water, the solvent becomes polar and the LDPE precipitates.^164^ After filtration to remove the precipitated polymer, CO_2_ and water is removed by mild heating to obtain the solvent in a non‐polar form for reuse.

For organic solvents, dissolution can be relatively slow owing to the small plastic/solvent contact area. Similarly, to solvolysis, the process could be accelerated by microwave heating (Figure 3 b) or ultrasonic irradiation drawing inspiration from related fields. Ultrasound assisted cellulose dissolution was achieved in ILs^165^ and starch dissolution in DMSO.^166^ Microwave‐assisted digestion has been described for analytical purposes of samples of PE, PP, PVC, and ABS in a mixture of hydrogen peroxide and nitric acid.^167^ In addition, breaking up the polymers into oligomers may also improve solubility in different solvents. This can be achieved through mechanochemical treatment, for example with exploding ultrasonic bubbles (Section 2.1.1).^82^

This approach in particular, but also other dissolution/precipitation techniques, yields a crystallized polymer that needs to be upgraded to obtain reliable virgin grade quality. Over its lifetime the polymer degrades through various mechanisms, such as photo‐oxidation and mechanical stress, leading to a reduction in chain length. The molecular structure, phase morphology, and mechanical properties of plastic waste streams can be improved by re‐stabilization, rebuilding of the macromolecular architecture, compatibilization of mixed recycled blends and addition of elastomers and fillers.^168^ In addition, the precipitated polymer has to be extruded to produce granulate to manufacture new plastic items.

More research is needed to obtain higher and more reliable recyclate quality. It could also be envisioned that the solvent/anti‐solvent system is expanded to separate plastic mixtures using several solvents that dissolve only certain type of polymers. In addition, further optimization of solvents and solvent/anti‐solvent combinations is critical. The solvent should:

1. selectively and strongly dissolve the desired polymer and no other added polymers or additives;
2. not be hazardous; and
3. easily evaporate for recovery and recycle.

### 2.4 New Avenues Towards Value Added Chemicals via Upcycling of Waste Plastic

One approach to overcome the often inhibitory low cost of virgin monomer is to convert waste plastics/polymers into useful, value‐added chemicals and/or materials (so‐called “Upcycling”).^169^ Whilst reports have described the use of waste plastic/polymers as the basis of, for example, transparent conducting films for photovoltaics,^170^ battery electrodes,^171^, ^172^ and carbon nanotubes,^173^, ^174^, ^175^ this Section will focus on the synthesis of chemicals and platform molecules.

Instead of depolymerization to monomers, plastics can be broken down into specific oligomers, which can be used, for example, as additives to tune the properties of inks and coatings for the printing industry making them glossier, easier to print out and more durable, a process patented and used by the company GreenMantra.^176^ Other reported examples of upcycling of polymers into more valuable materials include the synthesis of fiber‐reinforced plastics, via combination of depolymerized PET with renewably sourced, bio‐derived olefinic acids.^177^ Kamimura et al. described the treatment of Nylon‐12 and Nylon‐6 with supercritical CH_3_OH to produce methyl ω‐hydroxydodecanoate, a fatty acid ester derivative with potential antimicrobial agent applications with 85 % yield.^178^, ^179^, ^180^, ^181^ With regard to polyolefins, Hakkarainen et al. reported the selective conversion of HDPE wastes to a few well‐defined products, namely, succinic, glutaric, and adipic acid through microwave assisted acidic hydrolysis.^182^ The acids were then converted into plasticizers to be used in PLA processing.^182^ Succinic acid is highlighted by the US Department of Energy as a key platform for the bio‐economy market,^183^ while adipic acid is currently the most common dicarboxylic acid produced from petroleum refining^183^, ^184^ with the global market size estimated to be higher than 2.7 megatons per year at a price of above 1500 Euro/ton. The company BioCellection saw this business opportunity and developed a process with a dual‐catalyst system.^46^ Plasticizers can also be obtained from recycling PET using eutectic solvents as catalysts.^185^ 5 wt % of a choline chloride‐based eutectic solvent (ChCl/Zn(Ac)_2_) was used together with 2‐ethyl‐1‐hexanol as solvent to obtain 100 % conversion of PET with a yield of 84.7 % of the plasticizer dioctyl terephthalate.

Another approach for the production of higher value products from post‐consumer plastic is the post‐polymerization modification to yield polymers with altered adhesion or surface tension properties.^186^ For example, fluoroalkylation of polystyrene foam waste with photocatalytically generated electrophilic fluoroalkyl radicals was successfully achieved to induce a higher water repellence.^187^

### 2.5 Thermochemical Routes and Pyrolysis

Pyrolysis as applied to plastics is the thermal decomposition in an oxygen‐free environment into a mixture of products similar to the ones obtained by fractional distillation of crude oil, which range from refinery gasses, through gasoline/naphtha and diesel to immobile residues. Sharma et al. pyrolyzed HDPE plastic bags at 400 °C to produce a diesel yield of up to 47 wt %, with only the minimum oxidative stability and density failing ASTM D975 and EN 590 standards,^188^ making it highly suitable in blending for direct fuel. In contrast, monomer recovery rates for HDPE are typically below 40 wt %.^189^

Pyrolytic products can be used as fuel or converted into monomers through the processes similar to the established routes of producing monomers from crude oil, such as steam cracking. It would, however, be desired to produce monomers from pyrolysis directly. While this is almost impossible for PET and PVC, high monomer recovery can be achieved for polymethylmethacrylate (PMMA) and PS (Table 2). For PVC, the activation energy for the evolution of HCl is less than half of the energy required to subsequently break the polyene chains. Therefore, it is kinetically challenging to selectively cleave the C−C bonds required to recover the monomer.^190^ The temperature of maximum mass loss (*T*_MAX_) for HCl formation and C−C bond cleavage can be found in Table 2. The significant difference of the *T*_MAX_ at which the two phenomena occur can be used to pre‐treat MPW containing PVC, selectively removing HCl.^191^ Interestingly, this shows similarities to biomass pyrolysis in which the variable thermal stability of hemicellulose, cellulose, and lignin is exploited to selectively depolymerize the respective fractions.^192^

**Table 2:** Plastic type with indicative monomer recovery rates from single stage pyrolysis rounded to the nearest 5 %.^[a]^

Plastic type:	Maximum pyrolytic monomer recovery rate: [wt %]	Temperature of: [°C]		Product(s) with greatest yield:
		*T*_5 %_	*T*_MAX_	
PET	ca. 0^196^	400^197^	435^198^	Benzoic acid, CO, CO_2_ and solid organics^196^
HDPE	40 (780 °C, 1.3 s, –)^189^	415^[199] [b]^	460^199^	pyrolytic crude oil^200^
PVC	ca. 0^201^	270^202^	290^202^, 465^[202] [c]^	HCl, Benzene^201^
LDPE	40 (860 °C, 0.6 s, –)^203^	375^204^	460^204^	pyrolytic crude oil
PP	30 (650 °C, –, –)^205^ 45 (650 °C, –, –)^[205] [d]^	355^206^ in Ar	470^206^ in Ar	pyrolytic crude oil
PS	70 (500 °C, short, –)^207^ 85 (500 °C, short, –, vacuum)^208^	400^[209] [b]^	435^209^	Styrene^207^
PMMA	>95 (450 °C, short, –)^210^	260^[211] [b]^	360^211^	Methyl methacrylate^210^

[a] Temperatures at which 5 wt. % mass loss occurs (*T*_5 %_) and at which mass loss rate is the highest (*T*_MAX_) obtained from TGA results rounded to nearest 5 °C and measured at 10 °C min^−1^ in N_2_ unless stated otherwise. [b] denotes that value was interpolated from a graph. [c] denotes the second maximum mass loss temperature for PVC as there are two separate degradation regimes. [d] The reported total yield did not account for carbon deposits left in the reactor.

For polyolefins there is some scope for increasing monomer recovery. Some examples of promising approaches for monomer recovery from PP are two step catalytic pyrolysis which has achieved 35 wt % monomer yield,^193^ whilst an induction‐coupled plasma reactor was capable of generating a 75 wt % monomer yield^194^ (this value was stated in vol % and converted into wt % assuming ideal gas law). Research in a pilot scale facility (200–500 kg per run) found similar yields of ethylene (36 wt %) and propylene (18 wt %) from mixed polyolefin waste, suggesting that a direct plastic to monomer plant could be feasible.^195^ Commercial plastic pyrolysis plants (i.e. Plastic Energy, Petronas, Fuenix Ecogy, and Nexus Fuel) do not currently aim for the production of monomers, but for fuel. It should be noted that values can be difficult to compare among different references because of inconsistencies in reactor designs and methods of calculating yield.

**Figure 6:**
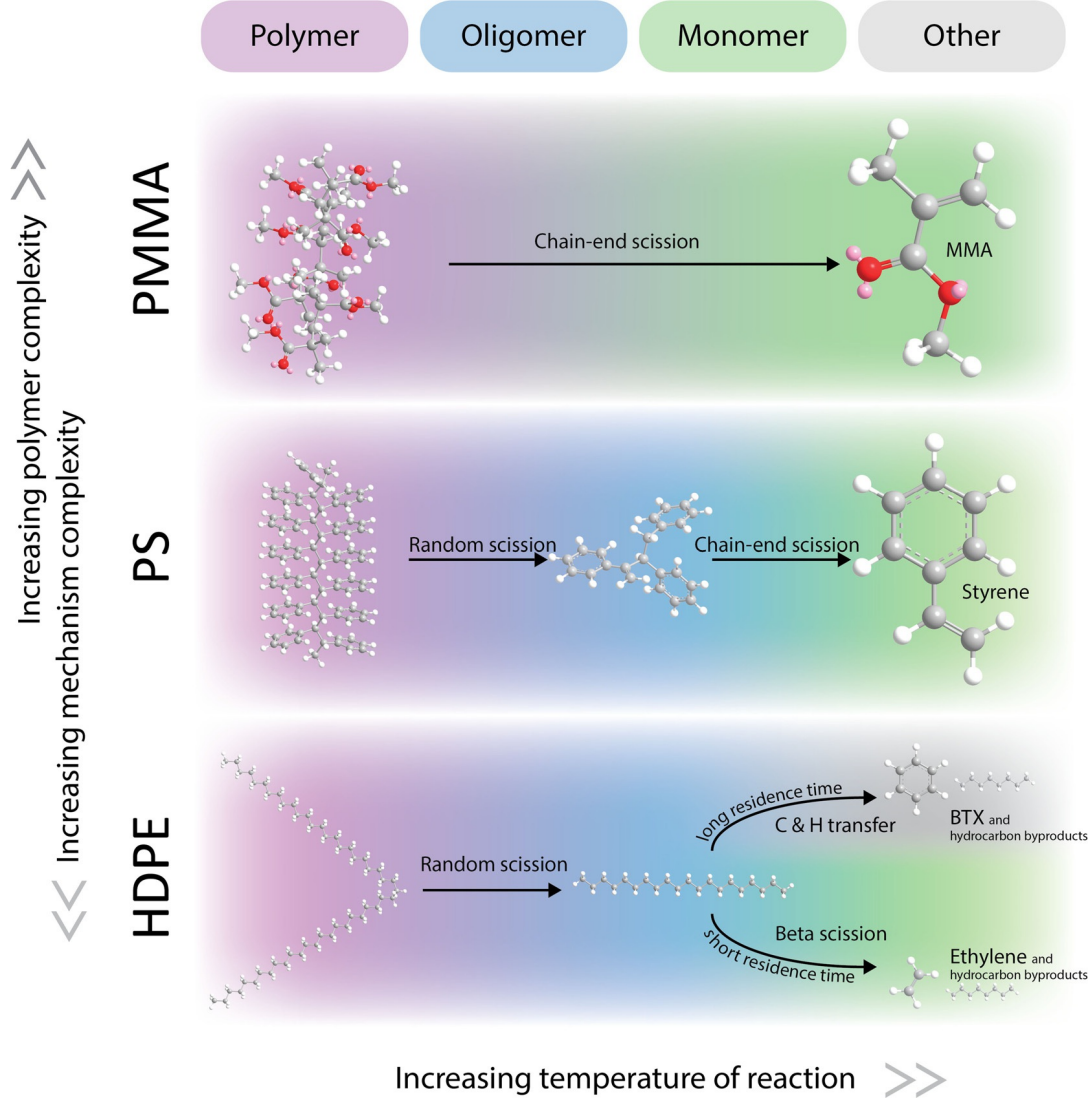
With an increase in the number of functional groups and heteroatoms in the backbone of the polymer (top), the distribution of products and the pyrolytic mechanisms become less complicated. Process parameters provide a higher degree of control over the product distribution for polyolefins, in this example HDPE, although the ultimate monomer yield is lower than for PS and PMMA. A common theme of all pyrolysis is that an excessively high temperature leads to coke formation given that the residence time is long enough. Although, most reaction steps occur at lower temperatures, similar trends are observed for catalytic processes. BTX denotes benzene, toluene, and xylene.

Yields of recovered monomer depend highly on reactor design (Section 2.5.4), because this influences temperature profiles and residence time. These two key parameters determine the product scope for pyrolysis, illustrated for HDPE and PS in Figure 6. Additionally, pyrolysis is typically performed at atmospheric pressure because of the increased coke and heavy fraction formation at elevated pressures.^212^

#### 2.5.1 Catalytic Pyrolysis

Catalysts have the potential to both reduce pyrolysis temperatures and narrow the product distribution.^213^ However, high viscosity, low thermal conductivity, and relatively long molecular chains lead to a small catalyst/polymer contact area, as well as inhibit heat and mass transfer.^10^ In some cases these issues can be overcome with novel catalyst design; for example, Pt nanoparticles were deposited on SrTiO_3_ nanocuboids through atomic layer deposition. The HDPE preferentially adsorbs onto the Pt, narrowing the product distribution without over cracking the products.^214^ A simpler, yet effective idea is nanocrystalline HZSM‐5 which exhibits an external surface area of up to 20 % of the total surface area, helping to overcome diffusion limitations.^215^ Catalyst properties and their influence on the reaction products are well summarized in the literature.^212^ Yet the search for a catalyst that is economical, selective, stable, and active enough to allow for industrial implementation is still ongoing.

#### 2.5.2 Reaction Mechanisms

With a focus on polyolefins, thermal pyrolysis proceeds through a free‐radical mechanism whereas catalytic pyrolysis proceeds through a combination of free‐radical and carbocation mechanisms. Subsequent reaction steps, inter/intra‐molecular hydrogen transfer and β‐scission, have different activation energy barriers and the prominence of one pathway over another can vary widely with small changes in temperature. Insight on the different pathways helps explain why product distribution changes with reaction conditions as seen in Figure 6. The key points are:

1. For polyolefins, initiation reactions are believed to occur through polymer chain imperfections rather than cracking of C−C bonds.^212^
2. The rate of random scission increases with the *M*_w_ of the polymer chain resulting in a tightening of the *M*_w_ distribution of the unreacted polymer through the reaction.^212^
3. β‐scission requires higher energy but produces shorter products than random scission in thermal pyrolysis, helping explain the increased yields of monomers at higher temperatures.^114^, ^216^
4. Longer residence times enhance secondary reactions, such as hydrogen and carbon transfer within carbocations, leading to cyclisation and aromatics formation.^212^
5. Stronger acid sites lead to a switch from random scission to chain‐end scission as the initiation step, which is preferred when aiming at shorter chain hydrocarbons. High monomer yields can be obtained from PMMA pyrolysis because of the dominance of chain‐end scission reactions as opposed to random scission in polyolefins.^212^

#### 2.5.3 Hydrocracking and other Reactive Gasses

Alternatives to N_2_ as a pyrolysis atmosphere (e.g. H_2_) can be used to tune product distribution. Hydrocracking is a promising process as it reduces coke formation through radical capping of the coke precursors and also operates at reduced reaction temperatures. These factors, in turn, prolong the catalyst lifetime.^212^, ^217^ The addition of H_2_ also creates a more highly saturated product (alkanes rather than alkenes) which can then be cracked using established steam‐cracker technology to form monomers for PP and PE synthesis. In addition to hydrogen and nitrogen, helium, argon, ethylene, propylene,^218^ and CO_2_ 219 have also been investigated. The use of a reactive gas reduces the coke formation and influences product yield. Pyrolysis in a CO_2_ rich atmosphere, for example, reduced the tar formation in PVC pyrolysis and reduced the acidity of pyrolytic oil for PET.^220^, ^221^ It was reported by Akah et al. that H_2_ also helps deal with any heteroatoms (e.g. Br and Cl) in the waste plastic,^222^ although handling of the generated acids may represent a challenge (e.g. with regard to reactor lifetime).

#### 2.5.4 Future Reactor Design

The choice of reactor is important for plastic‐waste depolymerization. For example, a fluidized bed reactor offers shorter reaction residence times, hence minimizing secondary reactions and side product formation. A reactor, such as a screw kiln, with longer residence times would typically be used for fuels and aromatics production. As discussed in Section 2.5, the following reaction parameters provide a handle for obtaining higher selectivity to desired products:

1. Heating rate
2. Temperature (distribution) of reaction
3. Solid/gaseous contact time
4. Gaseous residence time

These factors, in addition to the challenges of physically handling the waste, should be considered carefully when selecting a reactor design. In this context, a conical spouted bed reactor facilitates high heating rates (10^4^ °C s^−1^) resulting in high monomer recovery rates from PS.^207^ Inspiration can also be drawn from biomass pyrolysis using a gas‐solid vortex reactor^223^ to overcome the heat and mass transfer limitations of plastic. Microwave assisted pyrolysis has also been touted as a promising option.^224^ However, the low dielectric constant of plastics, requires microwave absorbers that can generate hotspots. Plasma assisted pyrolysis facilitates rapid heating to high temperatures, leading to reaction steps not typically observed in conventional pyrolysis and as a result achieves approximately double the monomer recovery rates.^194^

## 3 Concluding Remarks and Outlook

Plastic waste has developed into a pressing problem and society is approaching a critical turning point. From an industrial level, several start‐ups have been founded, and major polymer manufacturers are promising or taking action. The coming years will tell whether a real change can be achieved in plastic recycling. In this context, chemical recycling will play an important role as plastic use can only be decreased to a limited extent and better package design, that is, by avoiding composite and multilayer materials, can only facilitate recycling, while not eliminating the plastic waste problem. Reuse is only possible in very limited cases, while mechanical recycling leads to lower quality materials and cannot be applied to all plastic materials. However, any plastic recycling depends on the improvement of cleaning and sorting, for example, by smart and automated sensory systems. An example for this is the most recycled polymer, PET from bottles, for which efficient collection systems exist in some countries. There is a clear mismatch in the purity of researched ideal plastics (mixtures) and available real‐life plastic waste‐streams, which hampers commercialization.

In this Review, we have presented the chemical processes of solvolysis, dissolution/precipitation, and pyrolysis as well as novel ways of performing them, such as supercritical fluids, microwave reactors, mechanochemistry and biotechnology. Owing to their respective limitations and advantages, a mix of different chemical technologies will likely be needed to improve current recycling rates. The LCA analysis provides an initial ranking of the various technologies in terms of their potential to avoid CO_2_ emission. From this data we can conclude that polymers should be kept intact as much as possible during recycling, as this will reduce overall energy demand. This way leads to less energy required to break and reform chemical bonds. This is why dissolution/precipitation scores very highly in CO_2_ avoidance. The main problem for dissolution/precipitation is the use of hazardous organic solvents, but in the CreaSolv® Process, for example, these are fully recycled. The second‐best option is to produce a high purity stream of monomers, because they can directly be repolymerized without the need for an additional process step. Monomer recovery is typically the highest in solvolysis, but it can only be applied to polyesters and polyamides unless emerging techniques, such as ionic liquids or supercritical fluids, are used. Traditional pyrolysis is the least preferred chemical recycling option in terms of LCA, but advances in catalyst and reactor design could lead to pyrolysis that directly yields monomers. In addition, it was demonstrated that emerging technologies, such as plasma‐assisted pyrolysis, can produce monomers in very high yields. And even the more‐traditional pyrolysis processes can be very useful when integrated smartly into existing refinery infrastructure and is, according to our LCA, still advantageous over incineration with energy recovery. However, PVC and PU as well as PET and PA should be avoided in pyrolysis or the plastic stream has to be pre‐treated, if the pyrolysis oil is to be fed to a steam‐cracker.

Another option is to produce other high value products from polymers (i.e. BioCellection), which provides a good business case. From pyrolysis this could be a very pure stream of BTX (benzene, toluene, xylene). Diesel and gasoline are another option with a lower market price. Solvolytic routes provide fine chemicals with a very high market price. Tuning solvolysis as well as pyrolysis processes towards value‐added products also requires the use of stable and selective catalysts.

We conclude that the development of new or improved catalysts, which not only very active and selective, but also stable, is a very important factor in improving current solvolysis and pyrolysis processes. In pyrolysis products can be tuned to high BTX or to high monomer recovery. For example, Seo et al. improved the weight fraction of aromatics in the oil product from HDPE from <1 % to 59 % with a zeolite ZSM‐5 catalyst at 450 °C.^200^ In that respect further improvements also have to be made regarding plastic/catalyst contact. Some proposed solutions are dissolving plastic, for example, in the recycled vacuum gas oil fraction or in crude oil prior to feeding to the pyrolysis reactor, using supercritical fluids or mechanochemical conversion. A more even heat distribution also helps to achieve higher yields of monomers or other desired products. This can be achieved in microwave reactors, with supercritical fluids or by mechanochemical conversion.

In other words, the future is bright for chemists and chemical engineers to find new and improved processes for chemical recycling of a wide variety of commonly used plastic materials, and much progress can be expected in the years to come. On the other hand, these scientific and technological developments will have to go hand in hand with better policy frameworks and platforms connecting all the important stakeholders.

**Table 3:** Abbreviations

Abbreviation	Full name	Synonyms/IUPAC
ABS	Acrylonitrile butadiene styrene	
BFR	Brominated flame retardants	
BHET	Bis(2‐hydroxyethyl)‐terephthalate	Bis(2‐hydroxyethyl)‐terephthalate
BTX	Benzene, toluene, xylene	
DMT	Dimethyl terephthalate	dimethyl benzene‐1,4‐dicarboxylate
EG	Ethylene glycol	Mono ethylene glycol (MEG), ethane‐1,2‐diol
EU	European Union	
EoL	End of life	
HBCD	Hexabromocyclododecane	1,2,5,6,9,10‐Hexabromocyclododecane
HDPE	High density polyethylene	
HIPS	High Impact Polystyrene	
IL	Ionic liquid	
LCA	Life cycle analysis	
LDPE	Low density polyethylene	
M_w_	Molecular weight	
MHET	Mono‐(2‐hydroxyethyl)terephthalate	
MHETase	Mono‐(2‐hydroxyethyl)terephthalate‐digesting enzyme	
MPW	Municipal plastic waste	
PA	Polyamide	Nylon, Perlon
PE	Polyethylene	
PEF	polyethylene‐2,5‐furandicarboxylate:	
	PET alternative derived from bio‐2,5‐furandicarboxylic acid	
PET	Polyethylene terephthalate	
PETase	Polyethylene terephthalate‐digesting enzyme	
PLA	polylactic acid	
PMMA	Polymethylmethacrylate	Acrylic
PP	Polypropylene	
PP‐GF	Glass fiber reinforced polypropylene	
PS	Polystyrene	
PSW	Plastic solid waste	
PU, PUR	Polyurethane	
PVC	Polyvinyl Chloride	
TPA	Terephthalic acid	Benzene‐1,4‐dicarboxylic acid, PTA (Purified Terephthalic Acid)
TRL	Technology Readiness Level	
UPR	Unsaturated polyester resin	
WEEE	Waste electric and electronic equipment	
wt %	Percentage based on weight

**Table 4:** Process descriptions

Process name	Description
Mechanical recycling (also: secondary recycling)	Physical treatment of the plastic to achieve a consumer product from plastic waste. The most common mechanical recycling process involves melting and re‐extruding the plastic.
	
Chemical recycling (also: tertiary recycling, feedstock recycling)	Instead of merely physically transforming the shape and macroscopic properties of the plastic, chemical changes are made through breaking bonds. Often the goal is to depolymerize the polymers into monomers. These can be used to synthesize new polymers, but other chemical building blocks can result as well. Feedstock recycling is used to describe the recycling back to feedstocks used to make new polymers that is either monomers directly or a crude oil resembling product that can be fed to steam‐crackers to produce monomers.
	
Depolymerization	Breaking the bonds of the polymers to form monomers or oligomers. Often other side products form as well due to side reactions or interaction with a reactive medium present during depolymerization.
	
Thermochemical routes	Includes all processes (i.e. pyrolysis, hydropyrolysis, gasification) that break polymer bonds solely through the input of thermal energy. This can be achieved under inert (i.e. N_2_) or reactive (i.e. H_2_ or O_2_) atmosphere. These processes are most widely applied to polyolefins, but also studied for PS, PET, PMMA and impurities of other polymers.
	
Pyrolysis (also: thermolysis, thermal cracking, catalytic cracking, liquefaction)	During pyrolysis (‐lysis, Greek for dissociation) the chemical bonds of plastic are broken due to thermal energy. The plastic is heated under inert atmosphere (i.e. N_2_) until permanent gasses, liquids and waxes are formed. This process usually yields a very mixed hydrocarbon stream. This process is also denoted catalytic cracking or thermal cracking depending on whether a catalyst is used. Liquefaction refers to pyrolysis or hydropyrolysis under pressure.
	
Hydropyrolysis (also: hydrogenolysis, hydrocracking)	Thermal break‐down of plastic under H_2_ atmosphere. More specifically, hydrogenolysis refers to C−C bond cleavage followed by hydrogenation on a monofunctional metal catalyst. Hydrocracking refers to the same process on a monofunctional acid catalyst or on a bifunctional catalyst comprising a metal and acid site (bifunctional hydrocracking).
	
Solvolysis	Solvolysis is applicable to polymers with heteroatoms in their backbone and cannot be used to break C−C bonds. The solvolysis processes are named after the cleavage agent used and include hydrolysis, alcoholysis (glycolysis and methanolysis), phosphorolysis, ammonolysis and aminolysis. Ether, ester and acid amide bonds can be cleaved this way.
	
Dissolution/precipitation	In this process a plastic containing additives and impurities of other polymers or materials is dissolved. A solvent is chosen to selectively dissolve the desired polymer. Unwanted additives are filtered out and the desired polymer is precipitated. Strictly speaking dissolution/precipitation is not a chemical recycling process as usually no bonds are cleaved. However, since chemical fundamental knowledge is needed to understand the solvent/polymer interaction, solvent design and solvent recovery this process is covered in this perspective and is often considered chemical recycling.

## Conflict of interest

The authors declare no conflict of interest.

## Biographical Information

*Ina Vollmer graduated with a MSc.E.P (2015, US) in Chemical Engineering from Massachusetts Institute of Technology (US). In 2019, she completed her doctorate research on methane dehydroaromatization at Delft University of Technology (The Netherlands, with Prof. Freek Kapteijn) after which she went on to Utrecht University (The Netherlands), where she is currently studying the reaction mechanism of chemical recycling processes of plastics in the group of Prof. Bert Weckhuysen*.

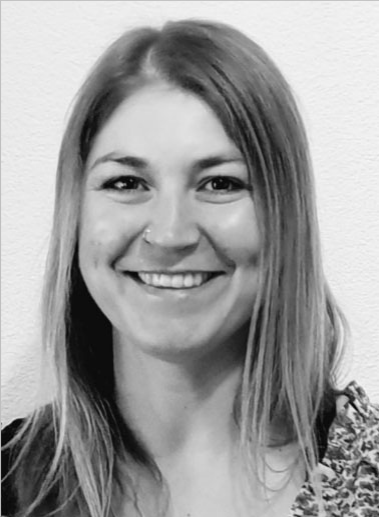

## Biographical Information

*Bert M. Weckhuysen obtained his PhD degree from K.U.Leuven (Belgium, with Prof.  Robert Schoonheydt) in 1995. After postdoctoral stays at Lehigh University (PA, USA, with Prof. Israel Wachs) and Texas A&M University (TX, USA, Prof. Jack Lunsford) in 2000 he became full Professor at Utrecht University (The Netherlands). His research focuses on the development and use of in situ and operando spectroscopy for studying solid catalysts under realistic reaction conditions at different length scales*.

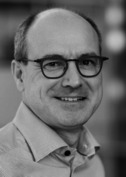

## Supporting information

As a service to our authors and readers, this journal provides supporting information supplied by the authors. Such materials are peer reviewed and may be re‐organized for online delivery, but are not copy‐edited or typeset. Technical support issues arising from supporting information (other than missing files) should be addressed to the authors.

Supplementary
