# Supplementary material for: Beyond Mechanical Recycling: Giving New Life to Plastic Waste
Source: Angew Chem Int Ed Engl. 2020 Jun 25;59(36):15402–23. doi: 10.1002/anie.201915651 (PMC7497176; doi:10.1002/anie.201915651)
Supplement: Supplementary file 1 — Supplementary [file ANIE-59-15402-s001.pdf]

Angewandte Chemie
Eine Zeitschrift der Gesellschaft Deutscher Chemiker

Supporting Information

Beyond Mechanical Recycling: Giving New Life to Plastic Waste

*Ina Vollmer, Michael J. F. Jenks, Mark C. P. Roelands, Robin J. White, Toon van Harmelen, Paul de Wild, Gerard P. van der Laan, Florian Meirer, Jos T. F. Keurentjes, and Bert M. Weckhuysen**

anie_201915651_sm_miscellaneous_information.pdf

Beyond Mechanical Recycling: Giving New Life to Plastic Waste

Ina Vollmer[a], Michael J. F. Jenks[a], Mark C. P. Roelands[b], Robin J. White[c], Toon van Harmelen[d], Paul de Wild[e], Gerard P. van der Laan[e], Florian Meirer[a], Jos T. F. Keurentjes[f], Bert M. Weckhuysen[a,*]

a. Inorganic Chemistry and Catalysis, Debye Institute for Nanomaterials Science, Universiteitsweg 99, 3584 CG Utrecht, The Netherlands
b. The Netherlands Organisation for Applied Scientific Research (TNO), Leeghwaterstraat 44, 2628 CA Delft, The Netherlands
c. The Netherlands Organisation for Applied Scientific Research (TNO), Materials Solutions Department, High Tech Campus 25, 5656 AE Eindhoven, The Netherlands
d. The Netherlands Organisation for Applied Scientific Research (TNO), Climate, Air & Sustainability Department, Princetonlaan 6, 3584 CB Utrecht, The Netherlands.
e. Energieonderzoek Centrum Nederland (ECN)- part of TNO, Biomass & Energy Efficiency, Westerduinweg 3, 1755 LE Petten, The Netherlands
f. University of Twente, Department of Energy Innovation, POB 217, 7500 AE Enschede, The Netherlands

E-mail: b.m.weckhuysen@uu.nl

Table of Contents

Table of Contents 1

S1 Summary of review articles 2

S2 Role of chemical recycling in a circular economy 8

S3 Life Cycle Analysis of Chemical Recycling Processes 34

S4 Analysis of Most Researched Processes and Plastics 36

S4.1 Method 36

S5 List of References 39

Table S1: Summary of review articles

Title: *(published date/ first available online)*	***Journal:***	***Resin identification code discussed:***	***Process:***	***Take away messages:***
**A review on pyrolysis of plastic wastes**[1]<br>(March 2016)	Energy Conversion and Management	1,2,3,4,5,6	Pyrolysis	- fluidised bed has the greatest economic potential for pyrolysis of plastic<br>- microwave-assisted pyrolysis offers benefits although inconsistent dielectric properties mean real waste streams are difficult to handle<br>- measurable impact of various carrier gasses with H_2 producing the least coke and Ar the most with the opposite trend in olefin production
**A review on tertiary recycling of high-density polyethylene to fuel**[2]<br>(May 2011)	Resources, Conservation and Recycling	2	Pyrolysis	- reaction pathways are temperature dependent highlighting importance in understanding heat and mass transfer limitations<br>- a wide variety of catalysts have been tested, each giving different product distributions
**Chemical recycling of plastics using sub- and supercritical fluids**[3]<br>(October 2008)	The Journal of Supercritical Fluids	1,2,4,7	Solvolysis	- supercritical 'solvents' allow for chemical recycling of certain crosslinked polyethylene (thermoset) without depolymerisation<br>- supercritical conditions can allow for almost 100 % monomer recovery from PET
**Chemical recycling of waste plastics for new materials production**[4]<br>(June 2017)	Nature Reviews Chemistry	1,2,3,4,5,6,7	Solvolysis, Pyrolysis	- Hurdles to commercialization are financial incentives and catalyst effectiveness<br>- Unique issues with each type of plastic highlighting the importance of reducing mixed polymer plastics<br>- Progress in design for recycling of polymers will facilitate chemical recycling
**Current state and future prospects of plastic waste as source of fuel: A review**[5]<br>(June 2015)	Renewable and Sustainable Energy Reviews	2,4,5	Pyrolysis	- generally for PO pyrolysis:<br>- thermal pyrolysis occurs through free radical mechanism<br>- catalytic pyrolysis proceeds through carbonium mechanism

				- importance of pre-treatment prior to pyrolysis for high quality fuel products
**Developing Advanced Catalysts for the Conversion of Polyolefin Waste Plastics into Fuels and Chemicals**[6]<br>(July 2012)	ACS Catalysis	2,4,5	Pyrolysis	- importance of accessibility of acid sites, promoted through large pore size or increasing surface area through decreased catalyst crystal size<br>- further study into deactivation and regeneration of catalysts need to be better understood<br>- two-step process holds great potential decoupling impurity removal/pre-processing with catalytically sensitive product formation<br>- large list of potential catalysts given
**Fuels from Waste Plastics by Thermal and Catalytic Processes: A Review**[7]<br>(October 2008)	Industrial and Engineering Chemistry Research	2,3,4,5,6	Pyrolysis	- reactor type and operating mode has large influence on product distribution due to heat and mass transfer limitations<br>- two stage processing results in better quality fuel<br>- the use of a solvent in the reactor can alter reaction mechanism and improve product distribution<br>- recirculation and use of pyrolysis gas as fluidising gas promotes BTX formation in 600 – 800 °C
**Hydrocracking of virgin and waste plastics: A detailed review**[8]<br>(April 2018)	Renewable and Sustainable Energy Reviews	1,2,3,4,5,6,7	Hydrocracking	- kinetics of hydrocracking and deactivation methods not well understood<br>- dependence on plastic type for optimum process conditions
**PET Waste Management by Chemical Recycling: A Review**[9]<br>(September 2008)	Journal of Polymers and the Environment	1	Solvolysis	- Polyethylene terephthalate (PET) polymer is difficult to purify once formed, so recycling needs to yield a very pure monomer to allow for repolymerization<br>- Large variety of PET available due to differing degrees of crystallinity<br>- Risks that legislation aims at eliminating polymers that have highest potential for recycling, like PET

**Plastics to fuel: a review**[10] (November 2015)	Renewable and Sustainable Energy Reviews	2,3,4,5,6	Pyrolysis	- work required to reduce costs associated with catalytic process<br>- heating rates of plastic impact the product distribution<br>- current legislation and economic driving forces do not create a market for plastic derived fuel oil
**Recycling and recovery routes of plastic solid waste (PSW): A review**[11] (July 2009)	Waste Management	1,2,3,4,5	Pyrolysis	- various recycling methods complement each other, there is no single recycling solution at this stage
**Recycling of waste from polymer materials: An overview of the recent works**[12] (October 2013)	Polymer Degradation and Stability	1,7	Solvolysis	- interesting suggestion that combining polymer types with 'compatibilizers' is the best option for mechanical recycling - how many rounds of recycling does this work for
**The valorisation of plastic solid waste (PSW) by primary to quaternary routes: From re-use to energy and chemicals**[13] (October 2009)	Progress in Energy and Combustion Science	1,2,3,4,5,6,7	Pyrolysis	- important to design future plastics with recycling (mechanical or chemical) in mind<br>- proper assessment of waste streams i.e. through LCA is vital to properly compare and develop waste processing techniques
**Thermal degradation of PVC: A review**[14] (December 2015)	Waste Management	3		- in HCl environment dechlorination is autocatalytic<br>- additives, especially stabiliser, can have a large impact on dechlorination process
**Thermochemical routes for the valorisation of waste polyolefin plastics to produce fuels and chemicals. A review**[15] (January 2017)	Renewable and Sustainable Energy Reviews	2,4,5	Pyrolysis	- Reactor design and process conditions crucial for tuning product distribution due to heat and mass transfer limitations in processing waste plastic<br><br>- Importance of (acid) catalyst for reducing reaction temperatures
**Thermolysis of waste plastics to liquid fuel: A suitable method for plastic waste management and manufacture of value added products—A world prospective**[16] (July 2009)	Renewable and Sustainable Energy Reviews	2,3,4,5,6	Mechanical, Pyrolysis	- future research should focus on more selective and regeneratable catalysts as well as real plastic waste<br>- mechanisms of depolymerisation summarised<br>- overview of process design considerations and impact on chemical recycling<br>- brief overview of some companies working in the field of chemical recycling of plastic

**Waste Polyolefins to Liquid Fuels via Pyrolysis: Review of Commercial State-of-the-Art and Recent Laboratory Research**[17] (April 2011)	Waste and Biomass Valorisation	1,2,3,4,5,6	Pyrolysis	- requirement for waste management legislation to keep up with waste production, processing methods and environmental targets<br>- issues for industrialisation include, catalyst coking, fouling, HCl above 200 ppm<br>- opportunities to crack plastic into suitable feed for industrial scale units
**Recycling of polyurethanes from laboratory to industry, a journey towards the sustainability**[18] (April 2018)	Waste Management	7	Solvolysis	- single-phase glycolysis of polyurethane yields a variety of monomers that do not allow for re-construction of flexible foams<br>- split-phase glycolysis provides potential for recovery of monomers for flexible PU as well, although currently only developed to pilot scale due to costs of cleavage agent
**Catalytic pyrolysis of plastic waste: A review**[19] (June 2016)	Process Safety and Environmental Protection	1,2,3,4,5,6	Pyrolysis	- geometrical limitations of catalysts result in wax formation on the surface of catalysts with smaller products (gasses) formed on the internal sites<br>- pore clogging is an important factor to consider with limited trials focussing on regeneration of catalyst<br>- deposits of impurities on the catalyst affect the activity but also remove these impurities from final product<br>- contains a table (Table 4) with various catalysts and their effect on the pyrolysis products<br>- investigation into cheaper and regeneration of catalysts to be investigated for industrial operation
**A review of polymer dissolution**[20] (July 2003)	Progress in Polymer Science	None specifically mentioned	Dissolution	- increased molecular weight results in decreased dissolution rate (chain disentanglement is a function of Mw)<br>- polydisperse samples have greater dissolution rate than monodispersed samples
**Solvent-based separation and recycling of waste plastics: A review**[21] (June 2018)	Chemosphere	1,2,3,4,5,6,7	Dissolution	- Gives details of strong and weak solvents for the various polymer types.

				- Solvent extraction from recycled polymer can cause damage to the polymer chain due to thermal stress.<br>- Dissolution of mixed polymer streams results in poorer separation of the target polymer.<br>- Future use of hazardous solvents should be reduced
**Mechanical and chemical recycling of solid plastic waste**[22]<br>(August 2017)	Waste Management	1,2,3,4,5,6,7	Mechanical, solvolysis, hydrocracking, pyrolysis	- Overview over both mechanical and chemical recycling methods with comparison of limitations, advantages and disadvantages of the different processes<br>- Degradation during mechanical recycling limits closed-loop recycling although mitigated through stabilisers and compatibilisers<br>- Design For Recycling and From Recycling are important in realising a circular economy for plastic<br>- Cl and N in waste stream deactivate catalysts in addition to inorganic components blocking pores<br>- Overview and analysis provided of various commercial projects and their status
**Catalytic co-pyrolysis of lignocellulosic biomass with polymers: a critical review**[23]<br>(May 2016)	Green Chemistry	1,2,3,4,5,6,7	Pyrolysis	- discussion of synergistic effects on the mechanism between biomass and plastics<br>- lists provided for results of non-catalytic (Table 2) and catalytic (Table 4) co-pyrolysis<br>- alkali metals from the biomass have significant impact on product distribution as they can catalyse the overcracking of the polymer chains
**Recycling of PVC wastes**[24]<br>(April 2011)	Polymer Degradation and Stability	3	Mechanical, pyrolysis	- usability of mechanically recycled PVC depends on its application (bottles performed very badly whereas pipes were acceptable)<br>- processes that chemically modify the PVC prior to recycling have been developed but are generally more expensive than mechanical recycling

				- pyrolysis of PVC usually involves a pre-processing step to remove HCl<br>- limited numerical data on the recycling and separation of PVC has halted the industrial implementation of new systems

Furthermore, interesting books in the field include:

- Feedstock Recycling and Pyrolysis of Waste Plastics: Converting Waste Plastics into Diesel and Other Fuels
- Material Recycling Trends and Perspectives

S2 Role of chemical recycling in a circular economy

Table S2. Companies and start-ups active in different fields of chemical recycling via dissolution/precipitation.

Type of process	***Process name***	***Company details:***	***Status:***	***Process description:***	***Patent:***	***Input:***	***product:***	***Country of operation:***
dissolution/ precipitation	Newcycling	**APK AG** www.apk-ag.de	commercial plant 2018, capacity: 8000 megatonnes/year, 2nd plant with 25000 megatonnes/year capacity planned for 2020		DE102016015197A1, DE102016015199A1 Solvent and method for dissolving at least two plastics from a solid within a suspension	multilayer films	pure PA, PE granulates	Germany
dissolution/ precipitation	CreaSolv® Process	**CreaCycle GmbH** https://www.creacycle.de/en/ partners: Fraunhofer IVV	Technology licensing and plant design	dissolution				Germany
dissolution/precipitation	CreaSolv® Process	**Fraunhofer IVV** https://www.ivv.fraunhofer.de/de/presseinformationen/circular-packaging.html partners: Lober GmbH & Co. Abfallentsorgungs KG, LÖMI GmbH		dissolution	WO2006131376A1, Method for recycling plastics and use thereof, WO2015000681 Method for increasing the concentration of at least one polymer from a polymer-containing waste material, and polymer recyclate 1997,EP0894818B1 Process for recycling soluble polymers or polymer blends from plastic containing materials	multilayer films PP/PET, PE/PA plus aluminium	pure polymers PE and PP	Germany
dissolution/precipit	CreaSolv® Process	**Lober GmbH/ LÖMI GmbH**	demonstration plant planned	dissolution		multilayer packaging film	polyolefins	Germany

dissolution/precipitation	CreaSolv® Process	**PolyStyreneLoop** https://polystyreneloop.org partners: partners: ICL-IP, BEWiSynbra, Fraunhofer IVV Members & supporters: +70 across the entire PS-value chain from all over Europe	Construction for demonstration plant in Terneuzen, the Netherlands started in December 2019 capacity: 3300 tonnes/year	dissolution		PS-foam (HBCD included) Current focus EPS, working on treatment of XPS	PS	NL
dissolution/precipitation	CreaSolv® Process	**Unilever** https://www.unilever.com/news/news-and-features/Feature-article/2018/our-solution-for-recycling-plastic-sachets-takes-another-step-forward.html partners: Creacycle	pilot plant in Indonesia	using CreaSolv technology for separation of PE multi-layer films		PE multilayer films		Indonesia
dissolution/ precipitation		**Polystyvert** http://www.polystyvert.com/en/ partners: Total	Technology licensing business with patents pending for process capacity: around 1000 tonnes/year assuming 8000 h operation	solvent cymene	WO2016049782A1 processes for recycling polystyrene waste	PS	PS	Canada
dissolution/ precipitation		**PVC Separation** https://www.pvcseparation.com/		delaminate multilayers by swelling the polymer in a low boiling solvent, followed by exposure to hot water, causing the solvent to flash and releasing the materials	WO2018035565A1 Separating polymer from composite structures	multilayer films		Australia

dissolution/precipitation		**Saperatec** https://www.saperatec.de/en/	operation of recycling plant mid 2021 capacity: 18,000 tonnes/year	reducing the interfacial forces of PET, PE and aluminium composites using separation fluids, for instance a micro-emulsion of an organic solvent, for swelling and a carboxylic acid for the acceleration of the separation to delaminate the films, the separation liquid is recycled	US20130319618A1 Separating Fluid, Method And System For Separating Multilayer Systems WO2015169801A1 Method and apparatus for recycling packaging material	multilayer films		Germany

Table S3. Companies and start-ups active in different fields of chemical recycling via solvolysis.

Type of process	***Process name***	***Company details:***	***Status:***	***Process description:***	***Patent:***	***Input:***	***product:***	***Country of operation:***
glycolysis	CuRE	**CuRE** https://curepolyester.com/about-cure/ partners: Cumapol, DSM-Niaga, DuFor and Morssinkhof, NHL Stenden University	pilot plant to be built in 2020, funding partners of the CuRe project are SNN, Province of Drenthe and the EU (EFRO)	PET chains are broken up to oligomers by glycolysis, followed by removal of impurities from the solution before repolymerization to longer chains		polyester	polyester granulate	
glycolysis	ChemPET	**Garbo** http://www.garbosrl.net/chempet-project/?lang=en	ChemPET project is funded by H2020			PET	BHET	Italy

glycolysis	VOLCAT	**IBM** https://newsroom.ibm.com/2019-02-11-IBM-Researchers-Develop-Radical-New-Recycling-Process-to-Transform-Old-Plastic		glycolysis at ~200 C under pressure, catalyst: volatile organocatalyst (1,5,7-triazabicyclo[4.4.0]dec-5-ene (TBD))	WO2015056377, Methods and materials for depolymerizing polyesters	PET	BHET	
glycolysis		**PerPETual** https://www.perpetual-global.com/ Adidas, H&M, Zara, Puma, Vero Moda and Decathalon	Ca. 2 million plastic bottles per day in a plant in Nashik, India	deconstruct PET chains to low Mw oligomers, followed impurity removal before repolymerization to longer chains	WO2013175497A1, Flakes of ester mixtures and methods for their production	PET bottles	Polyester yarn	India
glycolysis		**Ioniqa** http://www.ioniqa.com/	10 kilotonne/year plant in NL operational since 2019	dissolution in ionic liquids/catalytic glycolysis with ethylene glycol, catalyst: magnetic nanoparticle with an positively charged aromatic moiety and a negatively charged salt complex	WO2016105198A1 Improved reusable capture complex; WO2014209117A1 Polymer degradation	PET	BHET	NL
microwave assisted glycolysis	Demeto-process	**Demeto** https://www.demeto.eu/			WO2013014650A1 Method and apparatus for the recycling of polymeric materials via depolymerization process	PET	EG, TPA	

microwave assisted glycolysis	Demeto-process	**Gr3n** http://gr3n-recycling.com/	pilot reactor built in 2014		WO2013014650A1 Method and apparatus for the recycling of polymeric materials via depolymerization process	PET	EG, TPA	Switzerland
glycolysis		**Eastman** https://www.eastman.com/Company/News_Center/2019/Pages/Eastman-offers-innovative-recycling-technology-for-polyesters.aspx	engineering feasibility study		WO 2013025186 A1	polyester	monomers	USA
basic alcoholysis/ dissolution orswelling		**LoopIndustries** https://www.loopindustries.com/en/		catalyst: alkali metal, alkaline earth metal or ammonium hydroxide	WO2017007965A1, Polyethylene terephthalate depolymerization	PET	PET	

hydrolysis	Econyl	**Aquafil** https://www.greenbiz.com/article/better-recycling-through-chemistry	Carpet recycling plant in Arizona and Phoenix, depolymerization takes place in Slovenia, capacity carpet recycling plants: 16,000 tonnes/year used carpets	carpets are first separated into nylon 6 (35%), polypropylene (15%) and calcium carbonate (50%) and then the nylon 6 is depolymerized into caprolactam through steam hydrolysis	WO2014072483 Method and device for treating polymers	carpets or other nylon scrap	Caprolactam, which is then used to produce nylon 6 for new carpets	Italy
	LuxCR	**Teijin films** https://www.plasticstoday.com/recycling/big-names-plastics-develop-technologies-push-forward-circular-economy/110194803760589 Partners: DuPont		Contamination is removed during the process through a combination of monomer and polymer filtration units and by vacuum extraction, which runs for several hours at temperatures between 270o and 300oC		mechanically recovered PET flake	BHET	

Table S4. Companies and startups active in different fields of chemical recycling via pyrolysis.

Type of process	***Process name***	***Company details:***	***Status:***	***Process description:***	***Patent:***	***Input:***	***product:***	***Country of operation:***
pyrolysis		**Agilyx** https://www.agilyx.com/ partners: Ineos Styrolustion, Americas Styrenics, joint-venture Regenyx LLC together Chevron Phillips	opened chemical recycling facility in Tigard, Ore capacity: 10 tonnes of PS per day to be sold by Ineos Styrolution and Americas Styrenics, plans a second facility with Ineos		2019, US10301235B1	PS	styrene monomer	USA

pyrolysis		**Fuenix Ecogy** http://www.fuenix.com/ partners: DOW	fully functioning recycling plant in Weert, NL		2014, NL2015089A Rotary kiln and insufflator before		fuel	NL
pyrolysis		**Nexus Fuels** https://www.nexusfuels.com/	Commercial plant built, proven and now in full operation, capacity: 50 tonnes/day, 16-18 kilotonnes/year, Planning on next phases of construction of stand-alone plants				fuel	USA
pyrolysis		**Vadxx Energy LLC** https://vadxx.com/	currently raising additional funding		2015, WO2013123377A1 Dual stage, zone-delineated pyrolysis apparatus.	mixed plastics	gas, fuel, coke	
pyrolysis	ReOil	**OMV AG** (formerly Austrian Mineral Oil Administration) https://www.omv.com/en/blog/reoil-getting-crude-oil-back-out-of-plastic; https://www.chemicalprocessing.com/articles/2019/how-industry-tackles-plastics-plague/	test facility with 100 kg/hr plastic waste processing capability and 100 l/hr output	solvent (a fraction obtained from crude oil) assisted pyrolysis with solvent recycle, temp: 350-450 C, catalyst: no	CA2834807C	PP, PE, PS and low amounts of PET and PVC	pyrolysis oil	Austria

pyrolysis	TACOIL	**Plastic Energy** https://plasticenergy.com/press-release-sabic-signs-memorandum-of-understanding-with-plastic-energy-for-supple-of-recycled-feedstock/ partners: SABIC, Petronas	two chemical recycling plants in Seville and Almeria, operational since 2014 and 2017	catalyst: no	US20120261247A1	MPW	fuel	UK
pyrolysis		**VTT** https://www.vttresearch.com/media/news/vtt-and-the-city-of-nokia-are-planning-plastic-recycling-in-the-eco3-business-park-in-nokia	WasteBuster research project					
pyrolysis		**RES Polyflow** http://www.respolyflow.com/ partners: BP, AM WAX	vessel capable of handling 54 tpd. Currently looking to operate own facilities, long term looking to license technology capacity: 18000 tonnes/year (EU) assuming 8000 hr/yr operation	pyrolysis (with pre-sorting)		MPW	fuel	

pyrolysis		**Renewlogy** http://renewlogy.com/projects/ partners: Sustane Technologies, Renew Oceans	Working on various projects including the Nova Scotia and Pheonix projects with modular design for system to process 2721 tonnes/year. Nova Scotia has completed warm commissioning and is expected to be operational during 2019. In addition, demonstration plant in Utah, USA. capacity: 2700 tonnes/year (EU)			MPW	fuel	
pyrolysis		**Patpert** www.patpert.in	40 installations with plastic waste processing capacities, varying from 300 to 20.000 kg/day plastic waste and strives to roll-out technology in the Netherlands.	co-feeding plastic waste with a silica/alumina based cracking catalysts to the pyrolysis reactor at 350–360 °C and subsequent separation of other products from heavy wax fractions. These are fed to a secondary catalytic cracking, after followed by an integrated fractionation column equipped with a catalyst fixed bed of catalyst catalyst: silica/alumina based cracking catalysts	IN490MU2014 2015, Process of converting a polymer to hydrocarbon products	Plastic waste	fuel according to EN590-specifications	Netherlands/India

pyrolysis		**Pyrocrat Systems** https://www.pyrocratsystems.com/		catalyst: yes			50 to 90% of Pyrolysis oil (used as replacement to industrial diesel), 3 to 25% carbon black (used as replacement to coal powder in furnaces and boilers)	India
pyrolysis	P2O	**Plastic2Oil Inc.** http://www.plastic2oil.com/site/home	System scaled from lab - 1 ton - 20 ton scale (not time frame indicated) company moved to licensing its P2O technology in 2014 but returned to fuel production in 2018		2011, US20150001061A1, System and process for converting plastics to petroleum products	unsorted unwashed waste plastic (PO preferably) (PET and PVC not accepted)	fuel	
pyrolysis	RT7000	**Recycling Technologies** https://recyclingtechnologies.co.uk/	Selling units to process waste plastic into Plaxx oil - plan to have 1300 worldwide by 2027<br>capacity: 9000 tonnes/year waste	catalyst: no		waste plastic	Plaxx (fuels and waxes)	UK
pyrolysis		**Alunova Pryrolysis** https://www.alunova-recycling.de/home/				Aluminium containing waste	Aluminium and pyrolysis oil	Germany

pyrolysis		**Pyral** https://www.pyralag.com/				Aluminium containing waste	Aluminium and pyrolysis oil	Germany
pyrolysis		**Pryme** http://www.pryme-cleantech.com/technology/	Permit for plant received, Capacity: 40,000T/year	Catalyst: yes		Waste plastic (PS, PE and PP)	90% liquids (naphtha) and 10% non-condensable gases (based on weight and plastics)	
pyrolysis		**Pyroil** https://www.pyroil.nl/		Gas-phase fraction used for heating			Fuel for ships	NL
pyrolysis		**Clariter** http://www.clariter.com/	60,000 tons per year units	Continuous thermal cracking with multistage refining	WO2010049824A2 Apparatus and method for conducting thermolysis of plastic waste in continuous manner		Oil, solvent, waxes	Poland
pyrolysis		**SEPCO Industries** https://www.sepcoindustries.com/	20,000 kg per day				fuel	

Table S5. Companies and start-ups active in different fields of chemical recycling via novel types of pyrolysis.

Type of process	***Process name***	***Company details:***	***Status:***	***Process description:***	***Patent:***	***Input:***	***product:***	***Country of operation:***

microwave assisted		**Enval Ltd.** http://www.enval.com/	capacity: 2000 tonnes/year			plastic aluminium laminates	fuel	UK
tribochemical pyrolysis	catalytic tribochemical conversion	**Recenso GmbH** https://recenso.eu/de/plastic-conversion.html partners: BASF	pilot plant, capacity: type CTCmodul2019: 400 l/h with 7,200 operation hours per year<br>TRL: 6/7	catalytic tribochemical conversion, friction is used to improve conversion, temp: below 400 C, atmospheric pressure, produced halogens are neutralized by being converted to a salt, at the inlet of the reactor the plastic waste is mixed with 'startup' oil, which is the heavy oil fraction recovered from the products<br>catalyst: zeolite		SPW with 12 % humidity	29% aromatics, 21% n-paraffins, 14% olefins, 11% naphtenes & i-parrafins	
hydrothermal liquefaction		**RenaSci** https://www.renasci.be/en partners: BlueAlp, Petrogas, Mourik, Den Hartog BV	constructing a 120000 tonnes/year facility for processing of MSW in Oostende, Belgium, capacity: 120000 tonnes/year MSW	sorting and recycling via hydrothermal liquefaction	US20180010050A1 Method and system for transferring plastic waste into a fuel having properties of diesel/heating oil	MSW	EN590 diesel	
hydrothermal liquefaction		**BlueAlp** https://www.bluealp.nl/	Technology licencing and plant design	pyrolysis with molten plastic feed, recycle of long chain hydrocarbons	EP3247775A1 Method and system for transferring plastic waste into a fuel having properties of diesel/heating oil	PE (LD/HD) / PP / PB 68 – 97 wt%, PS / EPS 0 – 25 wt%, PVC: max. 2 wt%, PET: max. 5 wt%, Water: max. 20 wt%	fuel	

Table S6. Companies and start-ups active in different fields of upgrading of pyrolysis oil.

Type of process	***Process name***	***Company details:***	***Status:***	***Process description:***	***Patent:***	***Input:***	***product:***	***Country of operation:***
pyrolysis oil upgrading		**Petronas** https://plasticenergy.com/press-release-petronas-chemicals-signs-mou-with-plastic-energy/ partners: Plastic Energy	feasibility study					
pyrolysis oil upgrading	ChemCycle	**BASF** https://www.basf.com/global/en/media/news-releases/2018/12/p-18-385.html partners: Recenso GmbH	first batch of pyrolysis oil fed to the steam cracker at site in Ludwigshafen in October 2018	feeding oil to steam cracker at 850 C				
pyrolysis oil upgrading		**Dow** https://www.plasticsnewseurope.com/news/virgin-plastics-producer-dow-goes-circular partners: Fuenix Ecogy	pledged to incorporate at least 100,000 tonnes of recycled plastics in its product offerings sold in the European Union by 2025	feeding pyrolysis oil to crackers				

pyrolysis oil upgrading	Cat-HTR	**Neste** partners: Licella, ReNew ELP	aiming for an industrial-scale trial 2019 and eventually to process more than 1 million megatonnes/year of plastic waste by 2030	Crack product from Cat-HCR further to produce monomers				
pyrolysis oil upgrading		**SABIC** https://plasticenergy.com/press-release-sabic-signs-memorandum-of-understanding-with-plastic-energy-for-supple-of-recycled-feedstock/ https://plasticenergy.com/sabic-and-customers-launch-certified-circular-polymers-from-mixed-plastic-waste/ partners: Plastic Energy	memorandum of understanding signed with plastic-energy for plant in Geleen, NL. operation planned for 2021 (demonstration plant planned for 10,000-20,000 tonnes/year by 2022) SABIC also has many patents for conversion of MPW	Feeding TACOIL from plastic energy to their cracking units		virgin polymer	KSA	
pyrolysis oil upgrading	Polymer Energy technology	**MK Aromatics** http://www.mkaromatics.com/ partners: M/s.Harita NTI Ltd.	Operational plant in Tamilnadu		pyrolysis oil	various aromatic hydrocarbon solvents, aliphatic hydrocarbon solvents	India	

Table S7. Companies and start-ups active in supercritical polymer recycling, upcycling, design for recycling and enzymatic plastic degradation.

Type of process	***Process name***	***Company details:***	***Status:***	***Process description:***	***Patent:***	***Input:***	***product:***	***Country of operation:***
design for recycling		**Sustanetech** https://sustanetech.com/about	Operational facility in Nova Scotia, Canada, capacity: 70000 tonnes/year of MSW			MSW		Canada
design for recycling		**DSM-Niaga** https://www.dsm-niaga.com/what-we-do.html partners: Cumapol, Mattex, Lacom	Full production facility in Geleen, Netherlands but the recollection of carpets is still in testing phase	carpet that is more easily recycled than caprolactam made from nylon 6, DuPonts nylon 6.6 harder to depolymerize	2017, EP3475353A1	carpet		
design for recycling		**Cumapol** https://www.cumapol.nl/news/design4recycling-cumapet-l04-100-top-layer/ https://www.cumapol.nl/news/topdutch-region-is-closing-the-plastic-loop/ partners: DSM-Niaga						

upcycling		**BioCellection**<br>https://www.biocellection.com/		oxidative oligomerisation, <140 C, atmospheric pressure<br>catalyst: 2	pending	HDPE, LDPE	valuable chemicals (succinic acid, glutaric acid, adipic acid, pimelic acid, suberic acid, and azelaic acid)	USA
upcycling		**Green Mantra**<br>http://greenmantra.com/<br>partners: Ineos Styrolution	Pilot plant completed in 2014 for polyolefins processing. In 2018 started construction of demonstration plant for PS processing.	upcycling to produce waxes and additives<br>catalyst: yes	2015, US8664458B2<br>Method for producing waxes and grease base stocks through catalytic depolymerisation of waste plastics	polyolefin	'value-added' waxes as additives (various applications)	Canada
Enzymatic degradati		**Carbios**<br>https://carbios.fr/			WO2017198786 A1<br>A process for degrading plastic products	PET, PLA		France
supercritical	Cat-HTR	**Licella**<br>https://www.licella.com.au/cat-htr/<br>partners: ReNew ELP, Mura	plastic recycling done on pilot plant for 10 years already	supercritical water<br>catalyst: yes	US20130276361A1	MPW		Australia

supercritical	Cat-HTR	**ReNew ELP** https://renewelp.co.uk/ partners: Licella, Mura	commercial plant planned, capacity: 80,000 tonnes/year	residence time: 20-25 min; continuous; mixing; very good heat transfer, because heat is introduced internally through the supercritical water; energy recovery by using is for flash distillation; temperature and residence time are parameters used to tune product composition catalyst: yes	US20130276361A1 Processing of organic matter Additional patents filed but not yet in public domain- PCT application due for publication 2020	End of life plastics unsuitable for mechanical recycling. Composite mixed polymers and organic contaminated waste plastic streams. Waste is sorted prior in a separate facility using IR and optical sorting; PVC content kept below 1 %	Products are distilled into separate fractions; Naphtha, Distillate Gas Oil, Heavy Gas Oil, Heavy Wax Residue	UK
supercritical	process developed by	**PureCycle** https://purecycletech.com/ partners: P&G	As of Jan 2019 facility was under construction	remove odor and colour from waste plastic, dissolution of PP in supercritical butane followed by precipitation of PP upon lowering the pressure	WO2017003802A1 Articles of reclaimed polypropylene compositions	PP	purified PP	USA

Table S8. List of companies and start-ups active in chemical recycling that are referenced in the CLP report[25].

Company name:	Process type:
Agile Process Chemicals LLP	pyrolysis
AmberCycle (moral fiber)	biological decomposition
Anhui Oursun Environmental Technologies	pyrolysis
Aquafill	pyrolysis
Axens	solvolysis
Battery Resources	n/a*
Bioplasatech	biopolymer producer*
BioXycle	biological decomposition*
Biest	pyrolysis
ByFusion	mechanical mixing*
Cadel Deinking	delamination and additive removal
Climax Global Energy	pyrolysis (microwave)
Cogent Energy Systems	gasification*
Connora Technologies	dissolution*
EcoFuel Technologies	pyrolysis
Ecopek	purification
Envion	pyrolysis*
Equipolymers	polymer manufacture*
Esun	biopolymer producer*
Evrnu	mechanical recycling*
Fulcrum BioEnergy	gasification
FWD Energy	pyrolysis (microwave)*
GEEP (Global Electric Electronic Processing)	pyrolysis*
Generated Materials Recovery	n/a*
Genomatica	bio based intermediates producer*
Geo-Tech Polymers	purification
Golden Renewable Energy	pyrolysis
Green EnrviroTech Holdings	pyrolysis*
Illinois Sustainable Technology Center	purification and pyrolysis
Jeplan	solvolysis and pyrolysis
Jet Plastics	mechanical recycling*

Karlsruhe Institute of Technology	pyrolysis
MBA Polymers	mechanical recycling*
Modular Genetics Inc.	bio based intermediates producer*
Natureworks	solvolysis
New Hope Energy	pyrolysis
Next Generation	purification
Omnifusion	additive infusion*
Opus12	carbon dioxide to chemicals*
Origin Materials	bio based polymer producer*
P4SB	solvolysis*
perPETual	solvolysis
Polycycl	pyrolysis
Pyrowave	pyrolysis (microwave)
Quality Circular Polymers	mechanical recycling*
Re:NewCell	mechanical recycling*
Reclaimed EcoEnergy	purification
Resinate Materials Group	solvolysis
Resynergi	pyrolysis
RESYNTEX	solvolysis*
Sep-All	n/a*
Sierra Energy	pyrolysis
The Infinited Fiber Company Company	n/a*
The Pennsylvania State University	pyrolysis
Total Corbion	biopolymer producer*
TRASH2CASH	solvolysis
Tyton BioSciences	solvolysis
University of Massachusetts-Lowell	mechanical recycling*
University of Portsmouth	biological decomposition
University of Ulsan	purification
Valoren	n/a*
Klean Industries	pyrolysis*
Kyoto Institute of Technology	biological decomposition*

* Companies not assigned a process type in the CLP report were categorised based upon information on their website. Where process type is not explicitly clear, it is left unassigned.

Table S9. Interview transcripts with start-up companies

Question:	Saperatec	CreaCycle GmbH	Polystyvert	NeuxFuels	PolyStyreneLoop
**1) In a few words, can you describe the process that your company developed?**	Saperatec works on recycling solutions for multilayer composites. As a recycling company we develop separation fluids which work at the interface of multilayer materials and reduces the interfacial forces to delaminate individual layers from each other. An important criteria is that Saperatec does not dissolve a component of the composite and thus aim to reach close to 100% material recovery rates. The output materials of the process can directly integrated back in the same application field without energy intensive intermediate steps. Conclusively, saperatec process produces secondary raw material with high purity which has a positive carbon dioxide and energy footprint in comparison to virgin plastic material production.	From website: "The CreaSolv® Process does not fall into the classification "Chemical or Feedstock Recycling", because the chemical structure of the polymer chains remains unchanged, whereas chemical reactions produce other substances. The dissolution of plastics is a physical process, because the substance (plastic) only changes its physical state from solid to liguid, and this can also be reversed again. It is for this reason why the CreaSolv® Process has to be classified as "Physical Recycling"."	Polystyvert has developed an innovative and profitable process that allows all forms of polystyrene (PS) to be recycled. Following a unique dissolution, purification, and separation process, the regenerated polystyrene resin is of very high quality, allowing many applications to incorporate 100% recycled materials, at a lower cost than virgin resin.	Executive Summary: Nexus is an operational, commercially-scaled 50 Ton/day plant (first of many) converting waste plastics to feedstocks, which in turn are converted back to virgin plastics. (100% circular). Process is environmentally friendly (no wastewater or air issues), end-to-end business including software, front-end handling, all regulatory requirements, training, strategic pricing/positioning guided by financially-driven metrics. Versus others' Nexus is 1/3'd Capex/ton, 6x more efficient, 20% higher, quality yield, and profitable after paying for plastics, at lower crude-index pricing. Operational and economically proven, Nexus has been shipping tanker loads of offtake and has secured sources/stockpiles of plastics. Now shifting to rapid rollout of plants in US/Globally with ability to construct multi-100 ton/day plants on a jointly owned and operated basis with large strategic partners. (These technologies are not well suited for licensing at early stages given complex,	PSLoop is working on the CreaSolv® Technology which is important to know in advance is a dissolution process so we are not breaking down the polymer into monomer so it's different to chemical recycling technologies. It is a physical recycling process for the polymer chain is not broken. It was developed by CreaCycle together with Fraunhofer IVV. It was developed already some years ago. We recycle PS-foam (EPS and XPS) that comes to us in a compacted form via our members that function as a HUB/collection point. We then shred the material and add a solution that dissolves the polystyrene and allows to filter out any impurities. By addition of an anti-solvent the PS-gel is formed and the HBCD will be in the solvent. Any solvent remaining is distilled and reintroduced in the process. The PS-gel is dried and extruded. The HBCD sludge is further treated at the Bromine Recovery Unit (BRU) of ICL IP which recovers the elemental bromine and safely destroys the HBCD. We focus on the legacy

				resource needs) Nexus is located 20 min from Atlanta airport.	HBCD which was included as flame-retardant in PS-foam insulation applications from 1960 – 2015. HBCD is today classified as persistent organic pollutant. Incineration was the only treatment possible. PSLoop now offers a sustainable solution that preserves resources and closes the loop thus contributing to the circular economy.
**2) What is your business case?**	Saperatec is currently transforming from technology development to a recycling service provider (waste input material processed to secondary raw materials). Saperatec will sell secondary raw materials like recycled polyethylene which are obtained out of the Saperatec process.	The CreaSolv® Process is adapted to specific plastic waste streams by Fraunhofer IVV with CreaSolv® Formulations from CreaCycle. In case of a commercialization Fraunhofer IVV will license the technology and CreaCycle will supply the licensee with CreaSolv® Formulations as specified by Fraunhofer IVV.	Technology licensing business model	Recycling fails if not economic. Nexus is not a technology, but a business focused on resolving the plastics problem technically and economically on a sustained, scaled basis. Please see one-pager attached for more detail.	Start construction end of 2019, starting operation Q1 2021. Set up as a co-operative working with the whole PS value chain with 70 companies across 18 countries in the EU only take in PS from these companies. Current geographic focus for incoming material is in NL and Germany. Funding from EU, province of Zeeland, loans and contributions by members and supporters. It is more economical for companies to provide this PS to PSLoop rather than incineration. Sell product to members to produce new PS products.
**3) How close are you to break-even?**	Currently, Saperatec strives for industrialization of the technology, starting operation in mid 2021.	As Chemical Recycling the CreaSolv® Process is still in pilot stage with one pilot plant running and others to be built.		Operating profitability proven.	The XPS waste is a bit a more complicated because you also have blowing agents HCFCs which makes the XPS waste a hazardous waste. We are now in a working group working on pre-treatment technologies. Based on the Montreal protocol you have to capture the HCFCs with an efficiency of 95 %. Its then more lucrative to bring the

					hazardous waste to us than to incineration which is very expensive.
**4) Do you use any patented technology?**	We do not use any foreign technology which is protected by patents. In contrast, the saperatec technology is secured by patents.	Yes – our partner Fraunhofer as licensor does.	Polystyvert owns the recycling technology patents. Patent delivered in Canada and China. Notice of acceptance received for Europe, certificates will be received beginning 2020.	Nexus Intellectual Property protected by Trade Secret, not patents. Not our own, no. Pyrolysis was patented a long time ago (1960s) and patents have since expired.	CreaSolv® developed by CreaCycle and Fraunhofer. Fraunhofer is a partner of PSLoop. CreaCycle will provide the solvent for the plant.
**5) Can you provide some details on your process:**		From website: "On November 8, 2018 Unilever announced that the CreaSolv® Pilot plant is fully operational and they are ready to start examining the technical and commercial viability of this technology6). If successful the process will be commercialized and the technology will be made open source, available also to investors and competitors.<br>The CreaSolv® Plant is designed for high-quality polyethylene (PE) recycling, because 60% of the layers consist of this polymer. The recovered PE will be used for the production of new sachets. The energy consumption for the recycling of 6 kg PE is the same as for the production of 1 kg virgin polymer with the new technology, thus enabling a circular economy with a smaller environmental footprint. The facility currently processes approximately 3 tons sachet waste per day and Unilever invested approximately 10 Millionen Euros)."			
**a) feedstock and products**	Feedstock: industrial waste; products for the first industrial plant: composites of polyethylene (PE), aluminum, polyethylene terephthalate (PET)	PE	Feedstock: PS waste: mainly EPS, XPS, HIPS.<br>Products: recycled PS pellets	Feedstocks, plastics (2,4,5,6s) with tolerance for 1,3, 7s and other organics and inorganics. (critical to operating successfully).<br>Products – noncondensables (used in operation), crude middle distillate, wax, char. Crude and wax used as feedstock for virgin plastics production. Char sold as an	EPS and XPS with maximum 7% impurities (<3% water). Products are comparable to virgin PS although exact details will be determined once facility is operational. The whole CreaCycle® Process has been included in the UN Basel Convention as the Best Available Technology (BAT) in the Basel convention general technical guidelines. Product

				additive or heat source for others' operations.	distribution depends on the source of PS. The final application will be back in the same application like including in XPS and X-EPS
**b) throughput, production capacity**	18,000 t/a	700 t/a	Our demo plant is design for 125kg/h. We can design it depending on the customer demand (250 kg/h, 500 kg/h, 1000 kg/h)	50 Tons (US)/Day is one module. Plants are 100T and can be sized up as needed, operations are already at commercial scale. Numerous redundancies built in.	3300 tonnes/year produces 3000 tonnes/year of PS recyclate expecting about a 10 % loss in impurities<br>Studies from Germany and Netherlands show that sufficient material is available. Also in rest of Europe material is available.
**c) by-products/ unwanted products**	All fractions of the input material PE/Alu/PET will be products of the process. Most of the separation liquid will be recycled. The remaining chemical loss will be treated with state of the art and proven waste treatment technology in order to comply with all regulations.		Materials other than polystyrene; other polymers, other additives (ink, pigmentation for eg).	None. Nexus has a pre-cycling section of the plant before conversion, to remove undesired plastics, metals. No air or water issues since the process is closed loop.	HBCD sludge, inerts, (H)CFCs from XPS (removed prior to processing)<br>Carbon black or graphite in the material can stain the final product but as new insulation foams are grey this is not relevant.
**d) type of process**	Core process: liquid-based; pre- and after-treatment processes (like shredding, extrusion,…): all standard industrial processes	Dissolution	Dissolution/Precipitation/Separation<br>Yield: more than 90%	pyrolysis – closed loop, no incineration, no catalysts, run at atmospheric.	Pre-treatment to remove contamination<br>Compaction for efficient transportation, temperatures should not become too high to avoid possible breaking of the polymer chains<br>Physical dissolution process via the CreaSolv® Process at the PSLoop plant
**e) process conditions**	confidential		Industrial conditions.<br>A PLC automatizes and controls the recycling plant. Programming of the automatic machines allows adjusting quantities, temperature areas, and heating and cooling loops. Due to confidential reasons, I can't explain more regarding the		Operators and recyclers are experienced in handling PS and keeping polymer undamaged<br>HBCD is converted in the BRU at 1100 deg Cels into HBR and further converted into bromine and to be used for new brominated

			temperature process in the production line.		polymers for PS foam insulation as well
**f) catalyst details (if applicable)**	No catalyst is used.		N/A		
**6) Concerns/opportunities for the future of your system/company?**	Opportunity: Demand for ecological recycling solutions increases significantly by legal, end-consumer/costumer as well as business perspective. The market of PE/Alu/PET waste, which we focus first, exceeds the capacity of our first plant by multiple times.<br>Concerns: fluctuation of input quality	a. The unwillingness to attach an end-of-life cost ticket to produced polymer to make sure that plastic waste gets a value and sorting and recycling can be paid.<br>b. Concern: https://www.linkedin.com/pulse/lacking-recycling-technologies-60-our-plastic-waste-gerald-altnau/ High-quality recycling needs sophisticated sorting technologies.<br>c. Opportunity: https://www.linkedin.com/pulse/plastic-waste-pollution-visible-tragedy-commons-gerald-altnau/ If society would realize how bad it is they should also realize that only investing in recycling can reduce the price paid by all of us with our health.<br>d. Opportunity – the CreaSolv® Process is based on a physical process, thus allowing the recycled polymer to be re-used in the original application. Therefore this process falls into the category of "Physical Recycling" (like Mechanical Recycling) https://www.linkedin.com/pulse/what-high-quality-plastic-recycling-gerald-altnau/	Scaling up an industrial innovative process is a long-term timeline. It takes times and it is capital intensive.<br>Concerns for the system: optimization. We can always improve the process.<br>Opportunities: our main customers are currently in Europe.	Nexus believes analysis of these technologies should be on both technical AND economical fronts. There are many technologies, but often economics, and essential to supporting a viable business elements are ignored. Some technologies are isolated, ignoring upstream (sourcing feedstock) and downstream (quality specification) impacts and requirements. As a result, some plants have been built, even licensed, and then are unable to deliver. No one or two actions make pyrolysis successful technically/economically – requires 100s of actions done well. This is why Nexus has hardware, software, processes, training, regulatory approvals, engineering, bundled into a business eco-system driven by financial metrics. If need be, we invite you to visit Nexus (15 minutes from Atlanta airport) to see it under NDA, another area that is often over-looked.	Financing, getting everybody on board with a new process. 'breaking the status quo'<br>The greater opportunity is that we have support of the whole value chain and the political support, but of course now you're breaking the status quo. Introducing a new route that had gone to incineration for many years but also incineration plants are not too keen on taking PS so it's a favorable market for us to operate in. Despite the support we have you always face barriers or challenges which you have to overcome because we're the first ones to do it. For example the notification procedure we are now setting up a new system that no-one knows about it. Can you notify a route that is not yet existing. We are starting up so that natural to have the challenges there.
**7) What do you see as the major challenges in moving towards a circular economy?**	More generic methods like pyrolysis have problems to leverage the full recycling potential of a specific composite material. On the other	If authorities and governments will not put binding regulations on producers of polymers and plastic articles to cover the end-of-life treatment cost, cheap solutions will be preferred like syngas and energy recovery.	Feedstock issues. Collection and sorting issues.<br>Regulation, more implication of all the actor of the supply chain: from the producer of the material, to the consumer, including public	Consumer habits. There's an over-abundance of plastics but even with the best recycling programs, habits still need to be improved. Corporations as	Getting everybody onboard to dare to make the change. People have to leave their comfort zone of business as usual.

	hand, more sophisticated approaches like the Saperatec approach suffer at the moment from mixed input streams or fluctuating quality like in the post-consumer market (yellow bag, green dot). The simpler or standardized the waste stream – the higher will be the recycling rates for the waste and/or the higher the target orientation of the recycling technology like Saperatec ones.		authorities to induce more sustainable behavior.	well as packaging needs changing as well to allow for more recyclability.	
**8) What policies do you think need to be implemented (on an international or national level) in order make the circular economy of plastics a reality?**	The usage of secondary raw materials (recyclates) which have very often ecological benefits has to be in some way beneficial to the plastic processing industry. Oil as fossil resource is relatively cheap for years now so that company's do not change their processes because of economic points of views. At the moment wasting nature has no or only a small price (see emission trading) . Due to Saperatec positive carbon dioxide footprint, a carbon dioxide tax would have positive impact. Another approach would be a legal framework for design for recycling. There are many other	see number 7	Extended producer responsibility<br>Percentage of recycled content in product<br>Better collection and sorting for plastics<br>Carbon tarification	Collection incentives driven by brands and supporting gov'ts, packaging designs, and like other industry – clear, measurable goals. Education and an understanding there are costs/efforts to recycling, but done properly can lead to far greater environment and societal benefits.	Standardized analysis for 100 ppm HBCD – requirement for final product since there is currently no certified method for this.<br>In some countries there is an agreement that above 1000 ppm it has to be treated through incineration or PSLoop process and in other countries its set at 100 ppm.<br>Need to ensure that there is a market for the recycled product. Policies to stimulate the demand for recycled products in products would help.<br>Make incineration and landfilling even less attractive.

	policies which could lead to positive results.				
**9) In which country do you operate?**	At the moment Germany/Europe. It is planned to move to other countries/continents after our first industrial plant is running smoothly and also extend to other application fields.	We are located in Germany but we consider our business to be global.	Demo plant in operation in Canada (Montreal, Qc). Future licensee users in Europe.	Currently US, going global.	Netherlands and Germany, looking to expand across Europe following start-up of current facility. Already have contacts in France that are interested.
**10) Other**					4-5 FTE growing to 15-16 FTE by the end of 2020<br>HBCD levels:<br>EPS: 5000 - 10000 ppm<br>XPS: 6000 – 14000 ppm

S3 Life Cycle Analysis of Chemical Recycling Processes

Transport related emissions from waste transport to the EoL facility is based on the Netherlands:

- Municipal waste collection service:
- 50 km to sorting facility and municipal solid waste incineration (MSWI)
- Transport by >32 tonne lorry using EURO 6 (RER) fuel:
- 150 km from sorting/shredding to EoL treatment plant
- 50 km waste from EoL treatment to MSWI
- 700 km to consumer

Process energy consumption is estimated based on lab scale experiments (100 g) performed at TNO. The EcoInvent3[26] database was used to estimate CO_2 emissions from polymer production and packaging manufacture as well as multilayer and electronic products (**Table S9-10**). CO_2 emissions from electricity are estimated based on a majority fossil-based electricity mix of the Netherlands.

Table S10. Assumptions regarding material efficiency, product quality and energy consumption for the different EoL analysed in the life cycle analysis for the different plastic waste streams (1 tonne of plastic waste = 710 kg plastic). Process energy consumption is estimated based on lab scale experiments (100 g) performed at TNO. The EcoInvent3[26] database was used to estimate CO_2 emissions from polymer production and packaging manufacture as well as multilayer and electronic products.

EoL technology	Material efficiency [wt.%]	Product quality compared to virgin material [%]	Energy consumption [kWh/kg waste]
Incineration	-	-	negligible
Landfill	-	-	-
Energy recovery	21 recovered electricity<br>8 recovered heat	Electricity assumed to be high voltage Dutch market mix	Energy demands are incorporated in the energy recovery efficiency
Pyrolysis (PP-GF)	For the PP part:<br>78 oil<br>14 gas<br>8 solids/char (assumed to be used for heating through combustion)	The assumed products are heavy fuel oil, natural gas and glass fibre (GF)<br>25 GF	0.6 heating pyrolysis reactor, the remaining 8 are assumed to be recovered from combustion of char
Mechanical recycling	96 for all plastics, except PET 76	50 for all plastics, except PET 66	Electricity use:<br>0.004 shredder<br>0.2 extruder
Solvolysis	90 PET	100 PET	Electricity use:<br>0.004 shredder<br>0.2 extruder<br>0.012 pump<br>Steam use:<br>0.16
Dissolution	89 ABS/HIPS/PET-PE<br>75 PET<br>95 PP-GF	80 ABS/HIPS/PET-PE<br>100 PET<br>For PP-GF:<br>90 PP<br>50 GF	Electricity use:<br>0.004 shredder<br>0.2 extruder<br>0.012 pump<br>Steam use:<br>0.0308 dissolution vessel

Table S11. Main assumptions used in the life cycle analysis for the different plastic waste streams (1 tonne of plastic waste = 710 kg plastic).[26]

Polymer	Composition [wt.%]	Carbon content [%]	Lower Heating Value [MJ/kg]
ABS	98 ABS<br>2 carbon black<br>0.00109 tetrabromobisphenol A (TBBPA)	86	35.2
HIPS	100 HIPS<br>Low amount of deca-brominated diphenyl ether (DBDE)	92	37.8
PET	100 PET	63	23.0
PET-PE	58 PET<br>32 LDPE<br>10 ethylene vinyl alcohol (EVOH)<br>Layer thicknesses[µm]:<br>PET 125<br>LDPE 50<br>EVOH 25<br>LDPE 50	72	31.2
PP-GF	52 PP<br>46 GF<br>2 carbon black	47	17.7

S4 Analysis of Most Researched Processes and Plastics

S4.1 Method

For performing this particular analysis a script was developed using the programming language python (packages: json, requests, pandas, codecs, BeautifulSoup, glob, re, numpy, string, nltk). A keyword search in title abstract and author keywords '{chemical recycling} AND plastic' was performed using the Scopus application programming interface (API) yielding 369 initial results. From these initial results, keywords provided by the authors were extracted. The initial 369 research articles were filtered for reviews and the references provided in these reviews were used to extend the list of relevant research articles. For the list of research articles extended in that way, a full-text search was conducted using the Sciencedirect API with access through the network of the University of Utrecht. This way, full-texts of 474 research articles were obtained. and searched for the list of relevant keywords. The list of relevant keywords was compiled by filtering the author keywords for words ending in 'lysis' 'nation' and 'cracking' for process types. For polymer types words containing 'poly' were assembled and for polymer abbreviations words containing 'p' and being no longer than 4 characters were filtered. The visualization of the keyword cloud was performed in the open software gephi version 0.9.2 (gephi.org)[27] using the Yifan Hu[28] and the label adjust layout algorithms as well as filtering for connections with a weight of at least 4.

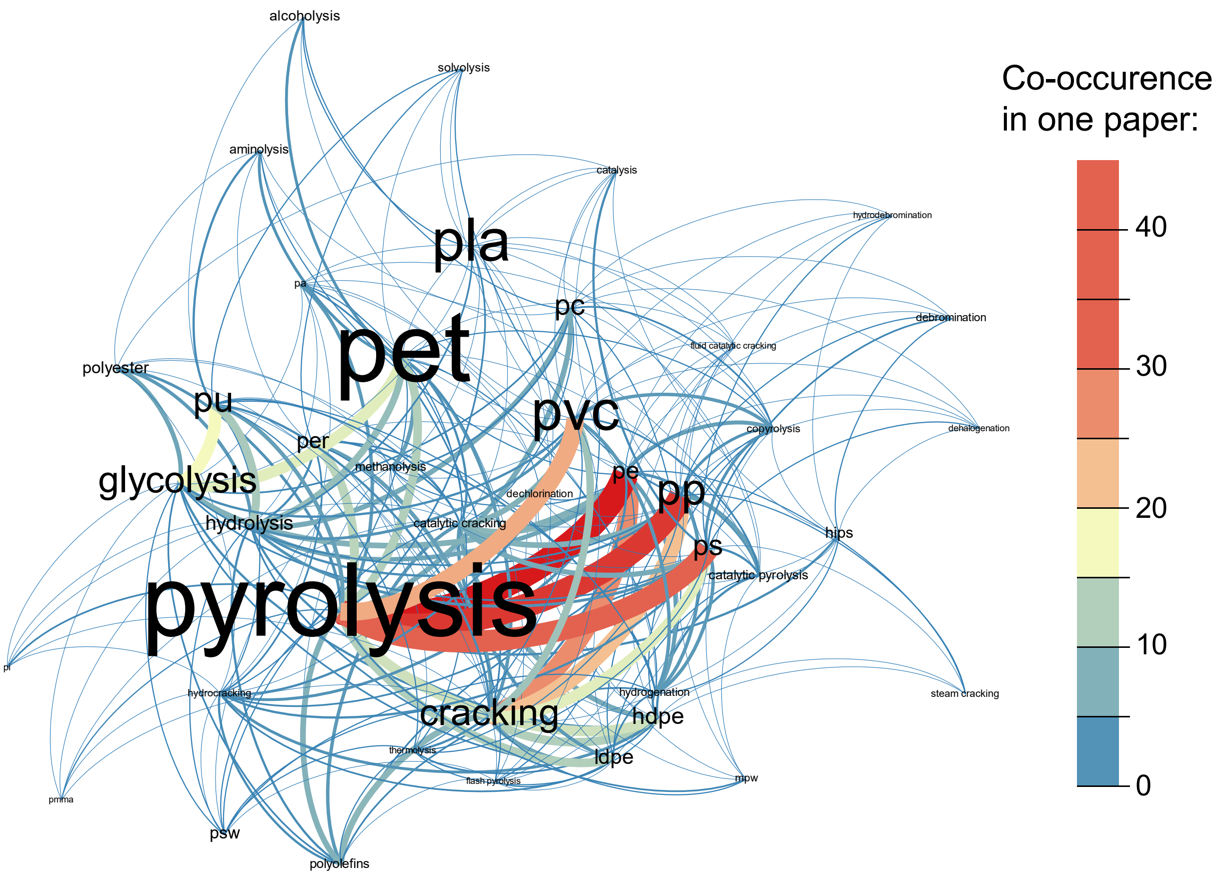

Figure S1. Keyword cloud generated to illustrate the most researched processes and plastic types as well as less researched areas. This keyword cloud generated by text-mining 474 articles, because they contained relevant keywords and were cited in relevant reviews. Keywords present author keywords filtered for plastic types and recycling processes. The thickness and colour of the connections between keywords present a measure for the number of times the two connected keywords were mentioned together in the same article. The font-size of the keywords represents how often they appear summed over all analysed journal articles. Occurrences of the acronyms and the long version of polymer names were summed and are presented as the acronym.

Table S12. Number of occurrences of the keywords depicted in the keyword cloud in **Figure 3** summed for all analysed articles.

keyword	occurrence
**pyrolysis**	2267
**pet**	2156
**pla**	1236
**pvc**	1166
**pp**	879
**glycolysis**	705
**cracking**	690
**pu**	662
**pc**	500
**ps**	498
**pe**	380
**hdpe**	353
**per**	334
**ldpe**	299

**hydrolysis**	279
**psw**	196
**polyester**	193
**hips**	155
**alcoholysis**	127
**aminolysis**	108
**polyolefins**	107
**catalytic pyrolysis**	104
**methanolysis**	83
**catalytic cracking**	79
**solvolysis**	70
**hydrogenation**	67
**debromination**	66
**mpw**	50
**catalysis**	47
**dechlorination**	46
**pa**	44
**upr**	43
**copyrolysis**	42
**hydrocracking**	34
**steam cracking**	34
**pi**	15
**pmma**	14
**fluid catalytic cracking**	12
**stepwise pyrolysis**	11
**dehalogenation**	10
**flash pyrolysis**	8
**peo**	8

**cracking catalyst**	7
**hydrodebromination**	4

S5 List of References

[1] S. D. Anuar Sharuddin, F. Abnisa, W. M. A. Wan Daud, M. K. Aroua, *Energy Convers. Manag.* **2016**, *115*, 308–326.
[2] S. Kumar, A. K. Panda, R. K. Singh, *Resour. Conserv. Recycl.* **2011**, *55*, 893–910.
[3] M. Goto, *J. Supercrit. Fluids* **2009**, *47*, 500–507.
[4] A. Rahimi, J. M. Garciá, J. M. García, *Nat. Rev. Chem.* **2017**, *1*, 0046.
[5] S. L. Wong, N. Ngadi, T. A. T. Abdullah, I. M. Inuwa, *Renew. Sustain. Energy Rev.* **2015**, *50*, 1167–1180.
[6] D. P. Serrano, J. Aguado, J. M. Escola, *ACS Catal.* **2012**, *2*, 1924–1941.
[7] J. Aguado, D. P. Serrano, J. M. Escola, *Ind. Eng. Chem. Res.* **2008**, *47*, 7982–7992.
[8] D. Munir, M. F. Irfan, M. R. Usman, *Renew. Sustain. Energy Rev.* **2018**, *90*, 490–515.
[9] V. Sinha, M. R. Patel, J. V. Patel, *J. Polym. Environ.* **2010**, *18*, 8–25.
[10] B. Kunwar, H. N. Cheng, S. R. Chandrashekaran, B. K. Sharma, *Renew. Sustain. Energy Rev.* **2016**, *54*, 421–428.
[11] S. M. Al-Salem, P. Lettieri, J. Baeyens, *Waste Manag.* **2009**, *29*, 2625–2643.
[12] K. Hamad, M. Kaseem, F. Deri, *Polym. Degrad. Stab.* **2013**, *98*, 2801–2812.
[13] S. M. Al-Salem, P. Lettieri, J. Baeyens, *Prog. Energy Combust. Sci.* **2010**, *36*, 103–129.
[14] J. Yu, L. Sun, C. Ma, Y. Qiao, H. Yao, *Waste Manag.* **2016**, *48*, 300–314.
[15] G. Lopez, M. Artetxe, M. Amutio, J. Bilbao, M. Olazar, *Renew. Sustain. Energy Rev.* **2017**, *73*, 346–368.
[16] A. K. Panda, R. K. Singh, D. K. Mishra, *Renew. Sustain. Energy Rev.* **2010**, *14*, 233–248.
[17] E. Butler, G. Devlin, K. McDonnell, *Waste and Biomass Valorization* **2011**, *2*, 227–255.
[18] D. Simón, A. M. Borreguero, A. de Lucas, J. F. Rodríguez, *Waste Manag.* **2018**, *76*, 147–171.
[19] R. Miandad, M. A. Barakat, A. S. Aburiazaiza, M. Rehan, A. S. Nizami, *Process Saf. Environ. Prot.* **2016**, *102*, 822–838.
[20] B. A. Miller-Chou, J. L. Koenig, *Prog. Polym. Sci.* **2003**, *28*, 1223–1270.
[21] Y. B. Zhao, X. D. Lv, H. G. Ni, *Chemosphere* **2018**, *209*, 707–720.
[22] K. Ragaert, L. Delva, K. Van Geem, *Waste Manag.* **2017**, *69*, 24–58.
[23] X. Zhang, H. Lei, S. Chen, J. Wu, *Green Chem.* **2016**, *18*, 4145–4169.
[24] M. Sadat-Shojai, G. R. Bakhshandeh, *Polym. Degrad. Stab.* **2011**, *96*, 404–415.
[25] *Accelerating Circular Supply Chains for Plastics*, **2019**.
[26] G. Wernet, C. Bauer, B. Steubing, J. Reinhard, E. Moreno-Ruiz, B. Weidema, *Int. J. Life Cycle Assess.* **2016**, *21*, 1218–1230.
[27] M. Bastian, S. Heymann, M. Jacomy, *Third Int. AAAI Conf. Weblogs Soc. Media* **2009**.
[28] Y. Hu, *Math. J.* **2005**, *10*, 37–71.
